# Inter-layer magnetic tuning by gas adsorption in π-stacked pillared-layer framework magnets†

**DOI:** 10.1039/d2sc06337a

**Published:** 2022-12-26

**Authors:** Wataru Kosaka, Honoka Nemoto, Kohei Nagano, Shogo Kawaguchi, Kunihisa Sugimoto, Hitoshi Miyasaka

**Affiliations:** a Institute for Materials Research, Tohoku University 2-1-1 Katahira, Aoba-ku Sendai 980-8577 Japan miyasaka@imr.tohoku.ac.jp; b Department of Chemistry, Graduate School of Science, Tohoku University 6-3 Aramaki-Aza-Aoba, Aoba-ku Sendai 980-8578 Japan; c Diffraction & Scattering Division, Japan Synchrotron Radiation Research Institute 1-1-1 Kouto, Sayo-cho Sayo-gun Hyogo 679-5198 Japan; d Department of Chemistry, Kindai University 3-4-1 Kowakae Higashi-Osaka Osaka 577-8502 Japan

## Abstract

Magnetism of layered magnets depends on the inter-layer through-space magnetic interactions (*J*_NNNI_). Using guest sorption to address inter-layer pores in bulk-layered magnets is an efficient approach to magnetism control because the guest-delicate inter-layer distance (*l*_trans_) is a variable parameter for modulating *J*_NNNI_. Herein, we demonstrated magnetic changes induced by the adsorption of CO_2_, N_2_, and O_2_ gases in various isostructural layered magnets with a π-stacked pillared-layer framework, 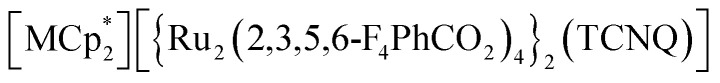, (M = Co, 1, Fe, 2, Cr, 3; Cp* = η_5_-C_5_Me_5_; 2,3,5,6-F_4_PhCO_2_^−^ = 2,3,5,6-tetrafluorobenzoate; TCNQ = 7,7,8,8-tetracyano-*p*-quinodimethane). Each compound had almost identical adsorption capability for the three types of gases; only CO_2_ adsorption was found to have a gated profile. A breathing-like structural modulation involving the extension of *l*_trans_ occurred after the insertion of gases into the isolated pores between the [Ru_2_]_2_–TCNQ ferrimagnetic layers, which is more significant for CO_2_ than for O_2_ and N_2_, due to the CO_2_-gated transition. While adsorbent 1 with M = Co (*S* = 0) was an antiferromagnet with *T*_N_ = 75 K, 1⊃CO_2_ was a ferrimagnet with *T*_C_ = 76 K, whereas 1⊃N_2_ and 1⊃O_2_ were antiferromagnets with *T*_N_ = 68 K. The guest-insertion effect was similarly confirmed in 2 and 3, and was characteristically dependent on the type of sandwiched spin in 
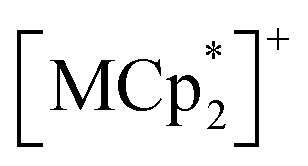
 as M = Fe (*S* = 1/2) and Cr (*S* = 3/2), respectively. This study reveals that common gases such as CO_2_, O_2_, and N_2_ can serve as crucial triggers for the change in magnetism as a function of variable parameter *l*_trans_.

## Abstract 2

The magnetic phase change triggered by structural modifications involving gas adsorption was realized for the first time in a layered metal–organic framework, where the inter-layer magnetic interaction was related to the inter-layer distance.

## Graphical abstract

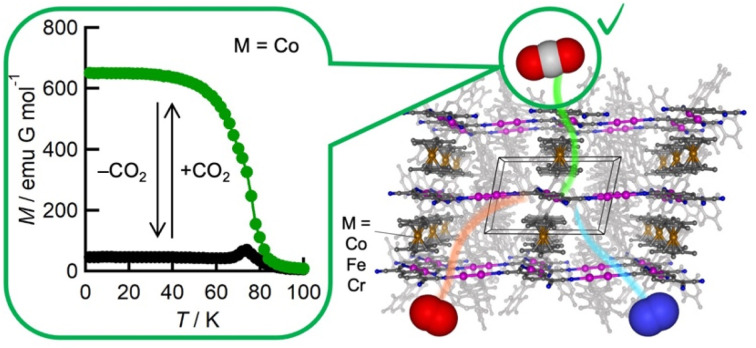

## Introduction

**Fig. 1:**
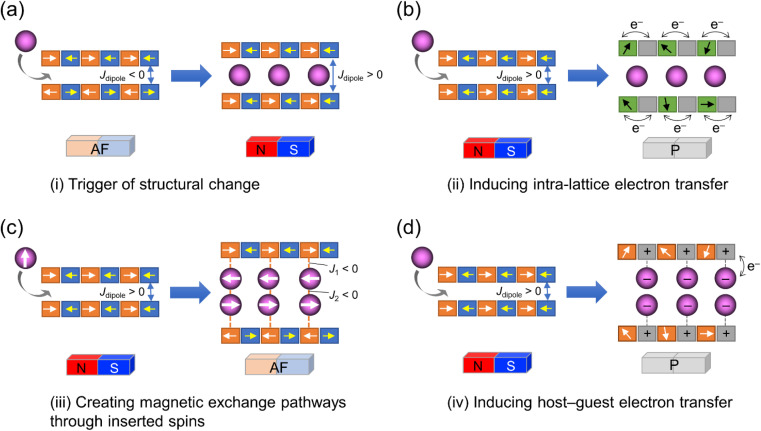
Four mechanisms for ON/OFF switching of layered magnets by guest adsorption. (a) Adsorption-induced structural change. (b) Adsorption-induced intra-lattice ET. (c) Creating magnetic exchange pathways through inserted spins. (d) Adsorption-induced host–guest ET.

The coupling of porosity-related capacity and magnetism is a fascinating approach toward the design of functional porous materials based on metal–organic frameworks (MOFs) and porous coordination polymers.^1–7^ Moreover, the focal points in the investigation of porosity and magnetism are fundamentally different. While the utilization of well-ordered pores of materials focuses on functionalizing the space bounded by frameworks,^8–15^ the concept of magnetism has mainly been utilized as a framework design to control spin ordering through the strong integration of paramagnetic spins through frontier orbitals of frameworks; this concept has also been applied to the design of magnetically conjugated paramagnetic frameworks.^16–21^ In contrast, the magnetism of low-dimensional framework systems such as layered MOF (LMOF) systems has gained considerable attention. In LMOF systems, inter-layer interactions, *i.e.*, through-space interactions, play a crucial role in tuning long-range magnetic ordering;^22–26^ hence, the absence of inter-layer interactions is considered evidence of mono-layer magnets.^27–31^ Therefore, magnetic LMOFs are a good platform for investigating guest effects in magnetism. Switching magnetic ground states, *i.e.*, the magnetic phases, as a function of the type of intercalated guest molecules between layers is a challenging subject.^32,33^ Indeed, guest-induced magnetic modifications of several magnetic LMOFs (bulk phase) have been reported so far, which are roughly categorized into four groups from the viewpoint of mechanism (Fig. 1a–d);^32^ (i) structural modification including crystal-amorphous change (Fig. 1a);^34–40^ (ii) induction of electron transfer (ET) in the frameworks (Fig. 1b);^41–45^ (iii) magnetic mediation by paramagnetic guest molecules (Fig. 1c);^46^ and (iv) host–guest charge transfer or ET (Fig. 1d).^47^ The modulation of the layer-stacking mode through guest adsorption/desorption is the subject of mechanism (i).^48–52^ However, the guest-induced variation in the magnetic ground state following mechanism (i) is only known in a few cases of magnetic LMOFs,^34–40^ and examples driven by common gases such as carbon dioxide (CO_2_), oxygen (O_2_), and nitrogen (N_2_) have not yet been reported.

**Fig. 2:**
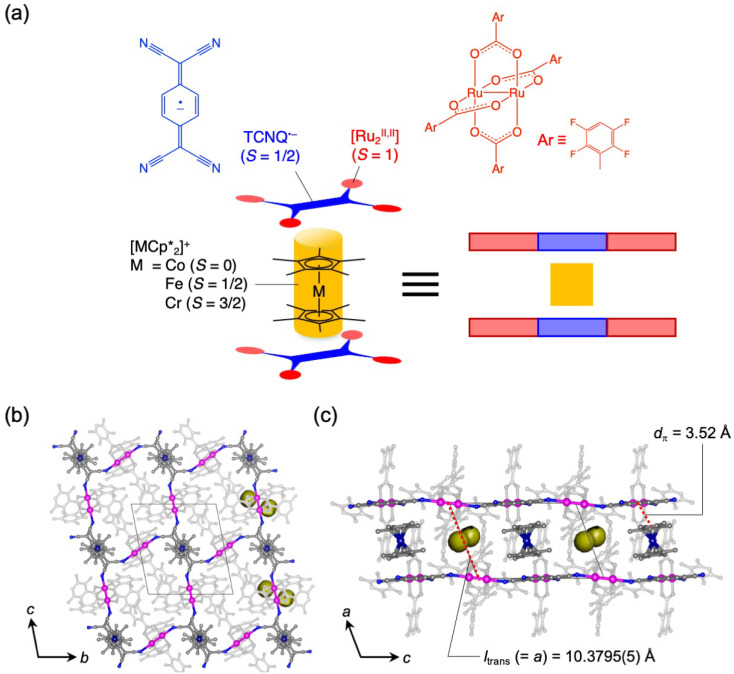
Structural overview of the series of π-PLFs and representative 1. (a) Schematic representation of the building units for the π-PLF. (b and c) Packing views of 1 projected along the *a*- and *b*-axes, respectively, where hydrogen atoms are omitted for clarity; C, N, Co, and Ru atoms are presented in gray, blue, navy, and purple, respectively; atoms of 2,3,5,6-F_4_PhCO_2_^−^ ligands are depicted in pale gray for clarity; and Connolly surfaces of void space are shown in yellow.

Herein, we report the gas-selective magnetic responsivity of a family of isostructural π-stacked pillared-layer compounds, 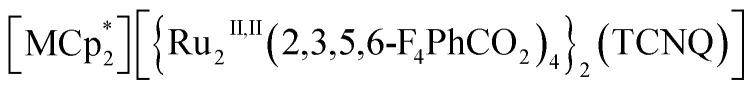 (M = Co, 1; Fe, 2; Cr, 3; Cp* = η_5_-C_5_Me_5_; 2,3,5,6-F_4_PhCO_2_^−^ = 2,3,5,6-tetrafluorobenzoate; TCNQ = 7,7,8,8-tetracyano-*p*-quinodimethane) (Fig. 2).^53,54^ These compounds are composed of ferrimagnetic layers [{Ru_2_^II,II^(2,3,5,6-F_4_PhCO_2_)_4_}_2_(TCNQ)]^−^ and sandwiched dodecamethylmetallocenium 
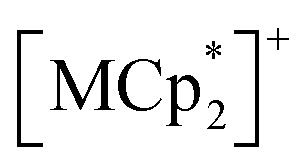
 in a π-stacking columnar mode as 
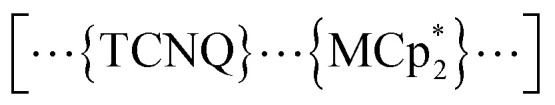
 (Fig. 2a),^55–60^ which are denoted as π-PLFs. The compounds have almost identical gas adsorption capabilities for common gases such as CO_2_ (4–6 mol mol^−1^ at 195 K), O_2_ (3–5 mol mol^−1^ at 120 K), and N_2_ (1–2 mol mol^−1^ at 120 K). However, they undergo a breathing-like structural modulation involving an extension of the inter-layer distance, which is more significant after the adsorption of CO_2_ than that of O_2_ and N_2_. Thereby, 1 with 
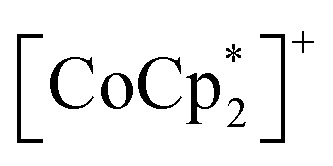
 (*S* = 0), which is an antiferromagnet (AF) with *T*_N_ = 75 K (*T*_N_ = Néel temperature),^54^ undergoes a change in the magnetic ground state to a ferrimagnet (F) with *T*_C_ = 76 K (*T*_C_ = Curie temperature) in 1⊃CO_2_, while maintains its antiferromagnetic state in 1⊃O_2_ and 1⊃N_2_ although their *T*_N_ is shifted to a lower temperature of 68 K than that in 1. The effect of guest insertion of CO_2_, O_2_, and N_2_ is similarly confirmed in 2 and 3, but is characteristically dependent on the type of the sandwiched spin in 
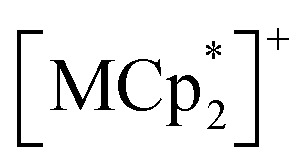
 with M = Fe (*S* = 1/2) and Cr (*S* = 3/2), respectively.^54^ As a typical prototype of mechanism (i), this work demonstrates that inter-layer magnetic interactions closely associated with inter-layer distance can tune the coupling of ubiquitous gas adsorption capability and magnetism in magnetic LMOFs.

## Results and discussion

### Gas adsorption properties of 1–3

Compounds 1–3 were synthesized according to a previous report.^54^Fig. 2b and c display the packing views of 1 as revealed by Rietveld analysis. In the π-PLF structures of 1–3, the lattice constant *a* corresponds to the inter-unit distance between two-dimensional [Ru_2_]_2_–TCNQ layers, also known as the translational distance, *l*_trans_, which was found to be 10.3795(5) Å (100 K), 10.2874(6) Å (135 K), and 10.5456(2) Å (120 K) for 1, 2, and 3, respectively (Fig. 2c). As shown in the Connolly surface views in Fig. 2b and c, small pores were present in 1 as isolated cavities surrounded by layers and 
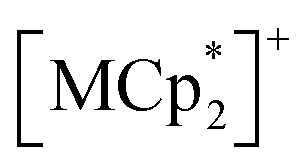
 cations.

**Fig. 3:**
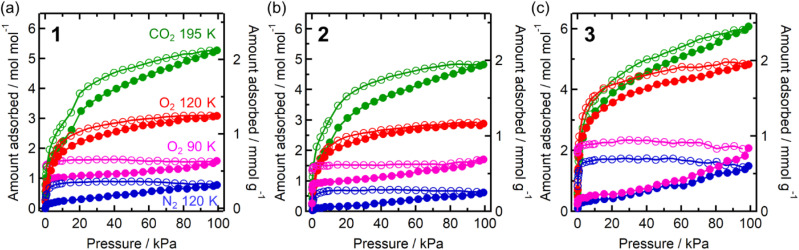
Gas adsorption (closed) and desorption (open) isotherms of 1–3 (a–c, respectively) for CO_2_ at 195 K (green), O_2_ at 90 K (pink) and 120 K (red), and N_2_ at 120 K (blue).

Each compound adsorbed different amounts of CO_2_, O_2_, and N_2_, but their adsorption/desorption isotherms were very similar (Fig. 3). Only 3 adsorbed slightly larger amounts of gases than 1 and 2, which could be due to its somewhat larger void space than those of 1 and 2 as realized in their pristine solvated compounds.^54^ At 99 kPa (= *P*_gas_), the amounts of adsorbed CO_2_ (195 K), O_2_ (120 K), and N_2_ (120 K) were 2.1 mmol g^−1^ (5.3 mol mol^−1^), 1.2 mmol g^−1^ (3.1 mol mol^−1^), and 0.3 mmol g^−1^ (0.8 mol mol^−1^) for 1; 1.9 mmol g^−1^ (4.8 mol mol^−1^), 1.2 mmol g^−1^ (2.9 mol mol^−1^), and 0.2 mmol g^−1^ (0.6 mol mol^−1^) for 2; 2.5 mmol g^−1^ (6.1 mol mol^−1^), 1.9 mmol g^−1^ (4.8 mol mol^−1^), and 0.6 mmol g^−1^ (1.4 mol mol^−1^) for 3, respectively. The amount of O_2_ adsorbed at 90 K was less than that adsorbed at 120 K. This could be because the adsorption is kinetically difficult at lower temperatures, such as 90 K, because the structure becomes more rigid.^43,46,61–63^ A small step was observed in the adsorption isotherm of CO_2_ at *P*_CO_2__ = 15–20 kPa for 1–3, albeit much more remarkable for 1 (Fig. 3). Despite being small, this step was an important sign that indicated a significant structural change in the process of CO_2_ adsorption (*vide infra*).

### Gas adsorption properties of 1–3

**Fig. 4:**
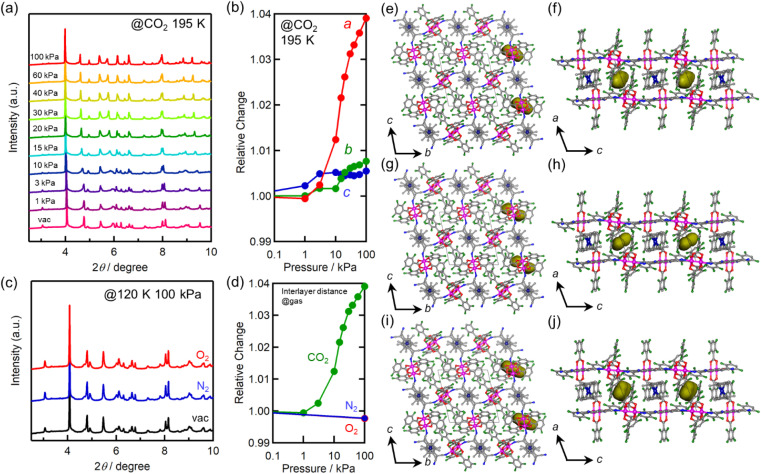
Structural changes of 1 upon gas adsorption. (a) CO_2_-pressure dependence of PXRD patterns measured at 195 K. (b) Relative changes in the *a*-, *b*-, and *c*-axes at 195 K as a function of CO_2_ pressure, where the values were normalized by the value under vacuum. (c) PXRD patterns at *P*_gas_ = 100 kPa of O_2_ and N_2_ and at 120 K. (d) The gas-pressure dependence of relative change of the *a*-axis for each gas. (e and f) Packing views of 1⊃N_2_ projected along the *a*- and *b*-axes, respectively. (g and h) Packing views of 1⊃O_2_ projected along the *a*- and *b*-axes, respectively. (i and j) Packing views of 1⊃CO_2_ projected along the *a*- and *b*-axes, respectively. In the figures of (e–j), hydrogen atoms are omitted for clarity; C, N, Co, and Ru atoms are presented in gray, blue, navy, and purple, respectively and Connolly surfaces that would be shared by accommodated gas molecules are shown in yellow.

*In situ* powder X-ray diffraction (PXRD) was conducted to gain insights into the crystal structures under gas adsorption conditions. The CO_2_-pressure dependence of the PXRD pattern of 1 at 195 K is shown in Fig. 4a. A small but distinct peak shift was observed after *P*_CO_2__ was increased from 10 to 20 kPa, corresponding to the step-like anomaly in the CO_2_ adsorption isotherm shown in Fig. 3a. The lattice constants obtained by Le Bail or Rietveld analysis for each pattern are summarized in Table S1,† and the changes in the lattice constants *a*, *b*, and *c* relative to those for pristine 1 are shown in Fig. 4b. Upon increasing *P*_CO_2__, the amount of adsorbed CO_2_ and, consequently, the lattice constants increased. Significantly, the lattice constant *a* (= *l*_trans_) increased non-linearly from 10.38 Å for pristine 1 (before introducing CO_2_) at *P*_CO_2__ ≈ 10 kPa, to 10.84 Å for 1⊃CO_2_ at *P*_CO_2__ = 100 kPa (total of +4% increase). In contrast, the increasing ratios of lattice constants *b* and *c* were less than 1%.

The PXRD patterns obtained under vacuum and at *P*_O_2_/N_2__ = 100 kPa at 120 K are shown in Fig. 4c, and the lattice constants obtained by Rietveld analysis are listed in Table S1.† The relative changes along the *a*-axis (= *l*_trans_) are shown in Fig. 4d. No significant change was observed in the PXRD patterns of gas-dosed 1⊃O_2_ and 1⊃N_2_. In fact, the length of the *a*-axis of 1⊃O_2_ and 1⊃N_2_ at *P*_O_2_/N_2__ = 100 kPa remained at 10.36 Å, which was comparable to that of pristine 1.

The structures of gas-adsorbed 1⊃CO_2_ at 195 K, 1⊃O_2_ at 120 K, and 1⊃N_2_ at 120 K were determined by Rietveld refinements using the corresponding PXRD patterns taken at *P*_gas_ = 100 kPa (Fig. 4e–j, Tables S1 and S2†), where the determined gas molecules are not displayed because of the inaccuracy of their local positions even in the Rietveld analysis. No significant difference was found in the frameworks of the gas-inserted phases (1⊃gas) when compared to those of pristine 1. Isolated gas-accommodating cavities with volumes of 155, 103, and 97 Å^3^ were found in 1⊃CO_2_, 1⊃O_2_, and 1⊃N_2_, respectively, located in the same position between the [Ru_2_]_2_–TCNQ layers and between the 
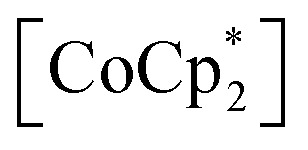
 cations regardless of the type of gas (but the amount was different). Their sharing positions are displayed as Connolly surface views in Fig. 4e–j.^54^ The π-stack distances (*d*_π_; see Fig. 2c), defined as the distance between the centers of the 6-membered ring of TCNQ and the 5-membered ring of 
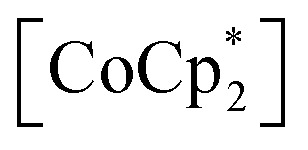
, were found to be 3.76, 3.49, and 3.51 Å in 1⊃CO_2_, 1⊃O_2_, and 1⊃N_2_, respectively. The values of *d*_π_ measured for 1⊃O_2_ and 1⊃N_2_ were very similar to that for 1 (3.52 Å), whereas the π-stack distance in 1⊃CO_2_ was 0.24 Å larger than that in 1 (Δ*d*_π_ = 0.24 Å). In fact, the change in *l*_trans_ between 1 and 1⊃CO_2_ (0.46 Å) is near twice the value of Δ*d*_π_.

Infrared (IR) spectra were recorded for 1⊃CO_2_, 1⊃O_2_, and 1⊃N_2_ at *P*_gas_ = 100 kPa and at 195, 120, and 120 K, respectively. The positions of the peaks corresponding to the C≡N stretching mode observed for 1⊃CO_2_, 1⊃O_2_, and 1⊃N_2_ remained unchanged compared to that for 1 (Fig. S2†), indicating no change in the electronic state after gas dosing.

### Magnetic properties of 1 under gases

**Fig. 5:**
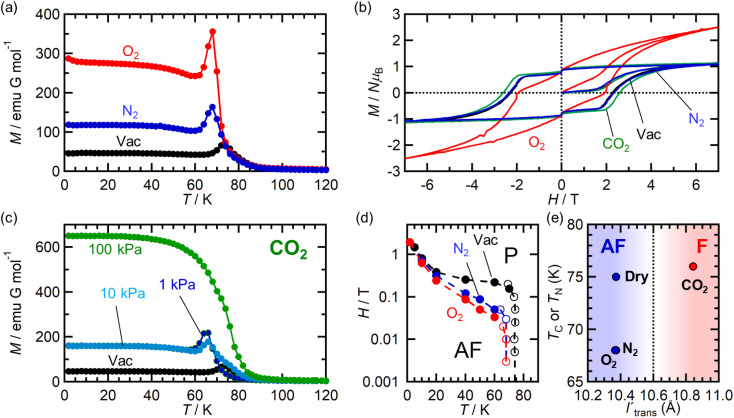
Variations of magnetic properties of 1 under N_2_, O_2_, and CO_2_ atmospheres. (a) FCM curves at *H* = 100 Oe for 1 measured under vacuum (black) and 1⊃N_2_ (blue) and 1⊃O_2_ (red) measured at *P*_gas_ = 100 kPa. (b) Magnetic hysteresis loops at 1.8 K for 1 measured under vacuum (black) and 1⊃N_2_ (blue), 1⊃O_2_ (red), and 1⊃CO_2_ (green) measured at *P*_gas_ = 100 kPa. (c) CO_2_-pressure dependence of FCM curves at *H* = 100 Oe for 1. (d) *H*–*T* phase diagrams for 1 (black), 1⊃N_2_ (blue), and 1⊃O_2_ (red), where AF and P represent the antiferromagnetic and paramagnetic phases, respectively. (e) Magnetic phase transition temperature (*T*_c_ or *T*_N_) *vs. l*′_trans_ plots for 1 and 1⊃gas (*P*_gas_ = 100 kPa).

Adsorbent 1 (*i.e.*, pristine form) is an AF with *T*_N_ = 75 K (black plot in Fig. 5).^54^ First, the behavior under a N_2_ atmosphere, that is, 1⊃N_2_, is described because 1 adsorbs only one molar equivalent of N_2_. When N_2_ gas at *P*_N_2__ = 100 kPa was loaded at 120 K, an adsorption equilibrium was reached to produce 1⊃N_2_. While this situation was maintained in a homemade cell at MPMS (Quantum Design Ltd.),^46^ the field-cooled magnetization (FCM) was measured under a magnetic field of 100 Oe (= *H*) (blue plot in Fig. 5a). The 1⊃N_2_ phase showed a cusp of FCM at 68 K (*T*_N_), indicating that it was still an AF. However, the *T*_N_ of 1⊃N_2_ decreased slightly in relation to that of 1 (Δ*T*_N_ = 7 K). Hence, 1 was affected by N_2_ accommodation, but not significantly. The magnetic field dependence of the FCM curves of 1⊃N_2_ exhibited characteristics of metamagnetism,^54^ showing a spin flip in the *H* range of 500–600 Oe (Fig. S3a†). This metamagnetic behavior was confirmed by varying the applied magnetic field (*H*) at a fixed temperature, where the initial *M* curve clearly displayed a spin-flipping phenomenon upon magnetic field sweeping (*M*–*H* curve, Fig. 5b and S3b†), easily recognizable in the d*M*/d*H vs. H* plots (Fig. S3c†). A *H*–*T* magnetic phase diagram was prepared based on these results (Fig. 5d). The *H*–*T* area of the AF phase in 1⊃N_2_ was smaller than that in 1. Although the AF phase of 1⊃N_2_ was very similar to that of 1 at low temperatures such as 1.8 K, the boundary between the AF and paramagnetic (P) phases was affected by the adsorption of only one molar equivalent of N_2_. Despite 1⊃N_2_ having the AF phase, it became a magnetic-field-induced F similar to 1,^54^ exhibiting the same magnetic hysteresis loop (Fig. 5b), where the saturated magnetization (*M*_s_) at 7 T was 1.1 *Nμ*__B__, the remnant magnetization (*M*_R_) was 0.9 *Nμ*__B__, and the coercive field (*H*_c_) was 2.35 T.

The magnetic behavior of 1⊃O_2_ was similar to 1⊃N_2_. O_2_ gas with *P*_O_2__ = 100 kPa was loaded at 120 K to address the adsorption equilibrium of 1⊃O_2_. In the FCM curve under *H* = 100 Oe, a cusp of FCM was observed at *T*_N_ = 68 K (red plot in Fig. 5a), indicating the transition to the AF phase. The *T*_N_ of 1⊃O_2_ was the same as that of 1⊃N_2_. The FCM curves of 1⊃O_2_ exhibited a spin flip in the *H* range of 200–300 Oe (Fig. S4a†), which was lower than those in 1⊃N_2_, suggesting that the AF phase in 1⊃O_2_ was more easily converted to the P phase than in 1⊃N_2_. By evaluating the spin-flipping fields from the initial *M*–*H* curve (Fig. 5b, S4b, and c†), an *H*–*T* magnetic phase diagram was prepared, as shown in Fig. 5d. The AF phase of 1⊃O_2_, as well as that of 1⊃N_2_, was regarded to be essentially identical to that of 1 at low temperatures (1.8–10 K). In the temperature range from 10 K to *T*_N_, the AF phase of 1⊃O_2_ is more easily governed by applying magnetic fields than those of 1⊃N_2_ and 1, likely in the order of 1⊃O_2_ > 1⊃N_2_ > 1. This order may be associated with the number of gas molecules accommodated.

In addition to 1⊃N_2_, 1⊃O_2_ is a magnetic-field-induced F with a magnetic hysteresis loop (Fig. 5b).^54^ However, the *M*_s_ value at *H* = 7 T of 1⊃O_2_ was 2.5 *Nμ*__B__, which is much larger than those of 1⊃N_2_ and 1. This is due to the paramagnetic contribution of accommodated O_2_ (*S* = 1) in the pores of 1⊃O_2_.^46^ However, it should be noted that the O_2_ spins could not be associated with long-range ordering mainly derived from the [Ru_2_]_2_–TCNQ layers; rather, the paramagnetic O_2_ contribution was likely superposed on the magnetic behavior of 1.^43^ Indeed, the value of *M*_R_ was the same (0.9 *Nμ*__B__) for all compounds. The *H*_c_ of 1⊃O_2_ was 1.95 T, which was only slightly smaller than that of 1.

The magnetic properties of 1 under the CO_2_ atmosphere were distinct from those under N_2_ and O_2_ atmospheres. The FCM curves of 1 were measured at *H* = 100 Oe under different CO_2_ pressure conditions (*P*_CO_2__ = 1, 10, and 100 kPa), after reaching adsorption equilibrium at 195 K (Fig. 5c). At *P*_CO_2__ = 1 and 10 kPa, 1 still exhibited an antiferromagnetic transition at *T*_N_ = 68 K, lower than *T*_N_ = 75 K for 1 and the same as those for 1⊃N_2_ and 1⊃O_2_. However, at *P*_CO_2__ = 100 kPa, where 1⊃CO_2_ is formed, the magnetization steeply increased at approximately 80 K and reached the maximum at 1.8 K without cusp or anomaly of FCM, indicating the onset of ferrimagnetic long-range ordering. This behavior was similarly observed even when a weak field, such as that at *H* = 5 Oe, was applied (Fig. S5†). Hence, 1⊃CO_2_ (*P*_CO_2__ = 100 kPa) was an F. *T*_C_ was determined to be 76 K from the *M*_R_*vs. T* curve (Fig. S5†). The *M*–*H* curve of 1⊃CO_2_ at *T* = 1.8 K was very similar to those of 1 and 1⊃N_2_, with *M*_s_ = 1.1 *Nμ*__B__, *M*_R_ = 0.9 *Nμ*__B__, and *H*_c_ = 2.62 T. The *H*_c_ value was slightly higher than those of the others (Fig. 5d).

Based on the magnetic properties of 1⊃gas, the state of 1 at *P*_CO_2__ ≤ 10 kPa could be identical to those of 1⊃N_2_ and 1⊃O_2_ (*P*_N_2_/O_2__ = 100 kPa). At higher *P*_CO_2__ values beyond the structural transition event at *P*_CO_2__ = 15–20 kPa, 1⊃CO_2_ could change to achieve the F phase. This implied that the magnetic phase of 1 was directly dependent on the amount of accommodated guest, regardless of the type of gas molecule (N_2_, O_2_, or CO_2_). Notably, all magnetic behaviors found under gases were entirely reverted to the original ones upon gas desorption by increasing the temperature to 350 K under vacuum (Fig. S6†).

### Magnetic phase change upon CO_2_ adsorption of 1

**Fig. 6:**
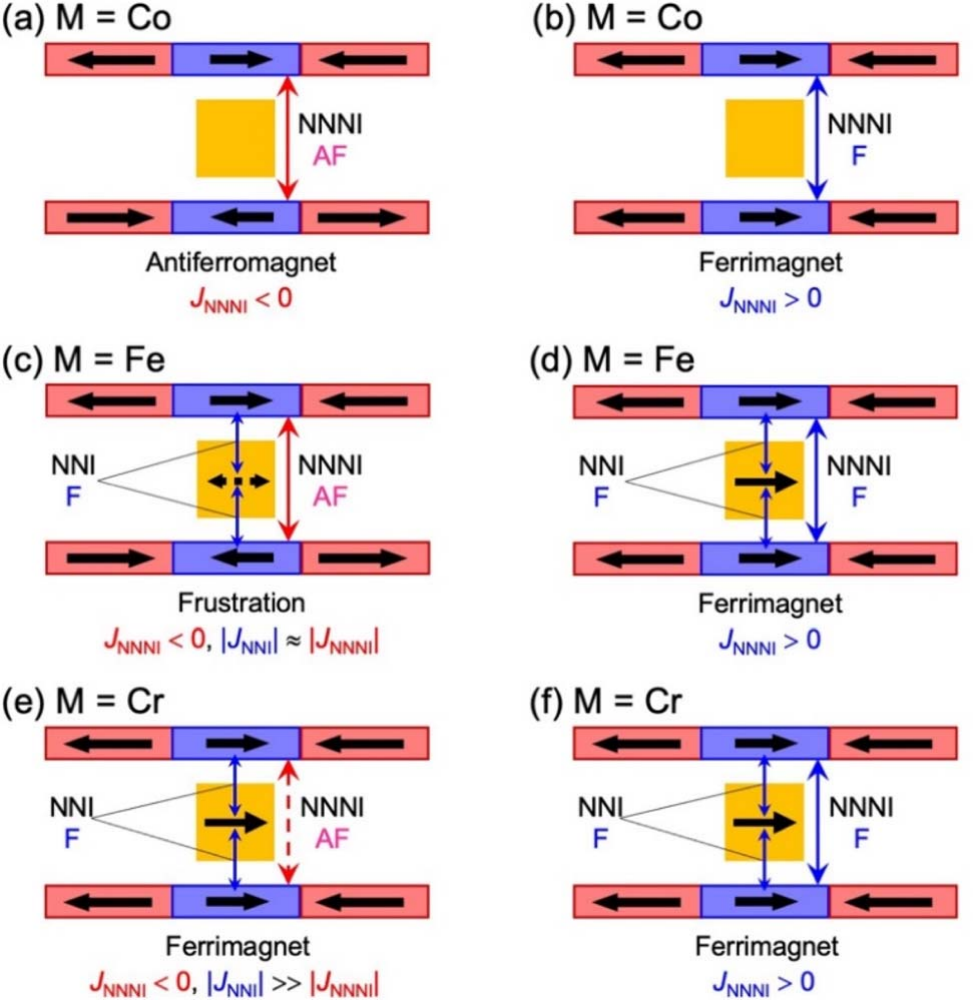
Schematic representation of the possible spin alignments in the present π-PLF.

Fig. 6 summarizes the possible spin alignments with a couple of magnetic interactions *J*_NNI_ and *J*_NNNI_ in the present π-PLF systems. *J*_NNI_ is the nearest-neighbor interaction, that is, the magnetic interaction between 
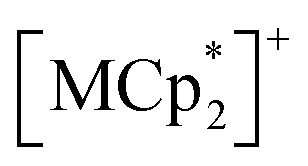
 and TCNQ˙^−^ radicals in the [Ru_2_]_2_–TCNQ layer, and *J*_NNNI_ is the next–nearest–neighbor interaction corresponding to the inter-layer magnetic interaction. Compound 1 showed a clear conversion from AF to F upon the complete adsorption of CO_2_ at *P*_CO_2__ = 100 kPa (*i.e.*, 1 changed to 1⊃CO_2_), whereas 1⊃N_2_ and 1⊃O_2_ were still AFs although their phases were distinct from that of 1 with a different *T*_N_. This change is likely due to the variation in the magnetic interaction between the two-dimensional layers *J*_NNNI_ because 
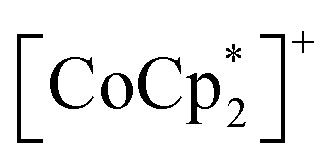
 is diamagnetic (Fig. 6a and b),^54^ which changes from AF to F upon CO_2_ adsorption. As an empirical rule, *J*_NNNI_ in the family of [Ru_2_]_2_–TCNQ layered magnets is related to *l*_trans_ as *l*′_trans_ = *l*_trans_/(cos *ψ*)^2/3^, where *ψ* is the angle formed between the Ru–Ru bond and a hypothetical flat layer of the [Ru_2_]_2_–TCNQ plane (Fig. S7†).^32,44,64,65^ When *l*′_trans_ < 10.6 Å, *J*_NNNI_ likely becomes AF.^32,44^Table 1 summarizes the relevant parameters in the series of 1 and 1⊃gas, and Fig. 5e shows the plots of magnetic transition temperature (*T*_C_ or *T*_N_) *vs. l*′_trans_. The *l*′_trans_ values of 1, 1⊃N_2_, and 1⊃O_2_ were all 10.37 Å, while that of 1⊃CO_2_ was 10.84 Å. These relationships demonstrate that only 1⊃CO_2_ was an F (Fig. 5e). The adsorbed amount of CO_2_ in 1 was higher than those of N_2_ and O_2_, which is a crucial reason more significant structural changes were induced only upon CO_2_ adsorption (Fig. 5e). Meanwhile, 1 at *P*_CO_2__ = 10 kPa was still an AF, similar to 1⊃N_2_ and 1⊃O_2_ (their *T*_N_ was also identical). Thus, the structural modification involving gate-type adsorption found around *P*_CO_2__ ≈ 15–20 kPa in the adsorption isotherm (Fig. 3) should be key for this transition. Indeed, the framework structure at *P*_CO_2__ < 15 kPa was identical to those of 1⊃N_2_ and 1⊃O_2_ (Fig. 4).

**Table 1:** Structural and magnetic properties of 1 and 1⊃gas

	1	1⊃N_2_	1⊃O_2_	1⊃CO_2_
*l*_trans_/Å	10.36	10.36	10.36	10.83
*ψ*/deg.	4.9	5.9	6.1	3.9
*l*′_trans_/Å	10.37	10.37	10.37	10.84
*T*_C_ or *T*_N_/K	75	68	68	76
Magnetic ground state	AF	AF	AF	F

Here, we comment on the effect of porosity on CO_2_ adsorption, which could be associated with CO_2_ diffusion at a higher temperature of 195 K. Compound 1 has isolated pores (Fig. 2a and b), and even all 1⊃gas compounds have isolated gas-accommodating pores. Thus, hopping from pore to pore is required for the gas molecules to penetrate deeply into a crystal. A high temperature, such as 195 K, could be favorable for transient structural modulations with the help of active thermal vibrations of the framework. Conversely, structural changes for hopping are unlikely to occur at low temperatures, which hinders the gas-hopping diffusion between isolated pores. For some gases at low temperatures, an adsorption equilibrium could not be reached within a practical measurement time because their gas diffusion was very slow. The O_2_ adsorbed amount in 1 (*P*_O_2__ = 99 kPa) was lower at 90 K than at 120 K, and N_2_ was adsorbed at 120 K but not at 77 K (Fig. 3). In addition, the kinetic molecular radius of CO_2_ (3.3 Å) is smaller than that of O_2_ (3.46 Å) and N_2_ (3.64 Å),^66^ which also facilitates diffusion and appears as a difference in the adsorption amount. Thus, both the amount of accommodated gas molecules and the type of accommodated gas are effective in this phenomenon.

### Magnetic properties of 2 and 3 under gases

Isostructural compounds 2 and 3 showed gas adsorption capabilities similar to that of 1 (Fig. 6) despite having different 
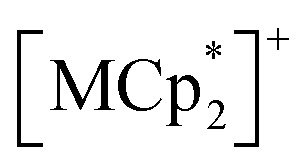
 moieties with M = Fe (*S* = 1/2) and Cr (*S* = 3/2), respectively.^54^ Therefore, the structural responses of 2 and 3 to gas adsorption were similar to that of 1 (details are described in the ESI†). Because only 3 has a slightly larger pore size with a larger *l*_trans_ (= *a*-axis) than 1 and 2 (see the section on structures), the amounts of adsorbed gases in 3 were slightly larger than those in 1 and 2 (Fig. 3). However, 2 and 3 have additional spins of 
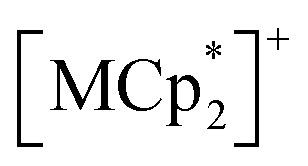
 with M = Fe (*S* = 1/2) and Cr (*S* = 3/2), respectively, between the [Ru_2_]_2_–TCNQ layers; therefore, *J*_NNI_ should be considered in addition to inter-layer interaction *J*_NNNI_ (Fig. 6c–f).

**Fig. 7:**
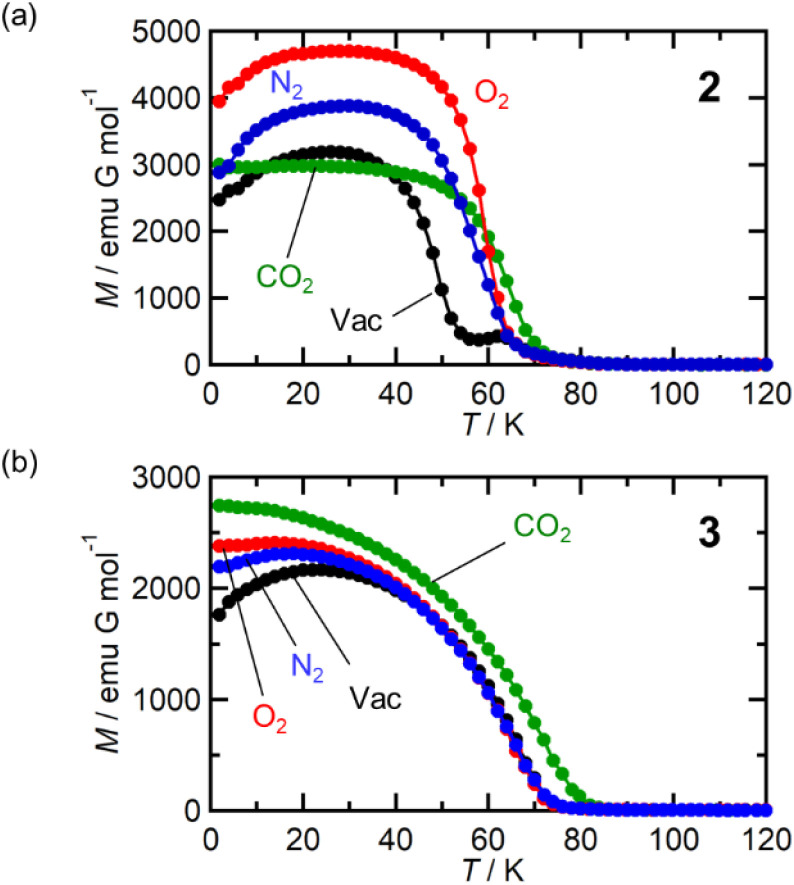
Variation of magnetic properties of 2 and 3 under gases N_2_, O_2_, and CO_2_. (a) FCM curves of 2 at *H* = 100 Oe measured under vacuum (black) and 2⊃N_2_ (blue), 2⊃O_2_ (red), and 2⊃CO_2_ (green) at *P*_gas_ = 100 kPa. (b) FCM curves of 3 at *H* = 100 Oe measured under vacuum (black) and 3⊃N_2_ (blue), 3⊃O_2_ (red), and 3⊃CO_2_ (green) at *P*_gas_ = 100 kPa.

Fig. 7a shows the FCM curve of 2 at *H* = 100 Oe under different gas atmospheres with *P*_gas_ = 100 kPa (Fig. S13† shows the remnant magnetization (RM), zero-field-cooled magnetization (ZFCM), and FCM. Fig. S14† shows the *M*–*T* curves of 2 at *H* = 5 Oe). Due to the competition between *J*_NNI_ (ferromagnetic) and *J*_NNNI_ (antiferromagnetic), as shown in Fig. 6c, 2 underwent a transition to a spin frustration phase (FR phase) at *T*_N_ = 69 K, followed by transfer to the F phase at 44 K (= *T*_R_) (Fig. 7a).^54,67–70^ Upon loading N_2_ at *P*_N_2__ = 100 kPa and 120 K, the FCM curve followed that of 2 but continuously increased without anomaly until *T* = 1.8 K, losing the FR phase under this condition (Fig. 7a). Namely, the antiferromagnetic interaction of *J*_NNNI_ in 2 could be weakened by N_2_ adsorption; consequently, the closest interaction *J*_NNI_ (ferromagnetic) for the π-contact 
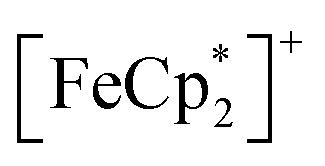
 and TCNQ moieties could be relatively dominant. The *T*_C_ value of 60 K was estimated for 2⊃N_2_ from the *M*_R_–*T* curve (Fig. S13†). Meanwhile, in the *M*–*T* curve of 2⊃N_2_ at *H* = 5 Oe, the *M*_R_–*T* curve showed a larger magnetization than the FCM curve in the temperature range of 30–50 K by passing through the quasi-field-induced ferrimagnetic state, indicating that the FR phase was maintained at a lower magnetic field of 5 Oe (Fig. S14a†). The behavior of “decreasing of the FR phase” was also seen for 2⊃O_2_, in which *T*_C_ was identical to that of 2⊃N_2_.

At first glance, the magnetic behavior of 2⊃CO_2_ appeared to be similar to those of 2⊃N_2_ and 2⊃O_2_ but was expected to have an F phase distinct from those of 2⊃N_2_ and 2⊃O_2_ (Fig. 7a). The *T*_C_ of 2⊃CO_2_ was 64 K, higher than that of 2⊃N_2_ and 2⊃O_2_. Instead, the whole feature of FCM in 2⊃CO_2_, representing the effect of CO_2_ accommodation in 2, was similar to that found in 1⊃CO_2_. Considering the similarity of the structure and gas adsorption capability between 1 and 2, *J*_NNNI_ in 2⊃CO_2_ and 1⊃CO_2_ were completely altered to ferromagnetic from antiferromagnetic in 1 and 2 (Fig. 6d).

The *M*–*H* curves of all 2⊃gas compounds were measured at 1.8 K (Fig. S15a†). The shape of the hysteresis loops was similar to that in 2, in which the anisotropic paramagnetic spin of 
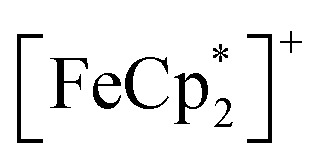
 was closely associated with the sigmoidal shape of spin flipping.^54,71^ In 2⊃O_2_ and 1⊃O_2_, the *M*_s_ values were larger than the others owing to the contributions from the paramagnetic spins of the adsorbed O_2_ molecules without involvement from the long-range magnetic ordering. The hysteresis loop in 2⊃CO_2_ was slightly modified with *M*_R_ = 0.90 *Nμ*__B__ and *H*_c_ = 1.76 T, proving that the state of the F phase in 2⊃CO_2_ was different from those in 2⊃N_2_ and 2⊃O_2_.

Fig. 7b shows the FCM curves of 3 recorded under different gases. Two types of cases exist on the spin alignment of pristine 3; because the contribution of *J*_NNNI_ (<0) should be much smaller than that of *J*_NNI_ (>0) (Fig. 6e), or because both key exchanges of *J*_NNNI_ and *J*_NNI_ basically induced ferromagnetism (Fig. 6f), 3 could be considered an F with *T*_C_ = 70 K.^54,72^ As expected, 3⊃N_2_ and 3⊃O_2_ showed FCM curves similar to those of 3, and a noticeable qualitative change in *T*_C_ was not observed. However, in 3⊃CO_2_, *T*_C_ became slightly higher (*T*_C_ = 78 K, from RM, Fig. S16 and S17†). The *M*–*H* curves measured at 1.8 K under N_2_ and CO_2_ were very similar to those of 3, which displayed a distorted hysteresis loop originating from the magnetic anisotropy of 
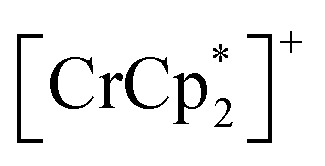
 (Fig. S15b†).^54^ Only 3⊃O_2_ revealed an O_2_-paramagnetic character superposed in 3. In addition to pristine 3, the closest ferromagnetic interaction *J*_NNI_ > 0 between the spin of 
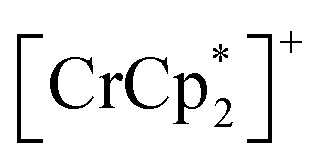
 (*S* = 3/2) and the TCNQ moieties of the [Ru_2_]_2_–TCNQ ferrimagnetic layers governed long-range magnetic ordering even under the gases.^54^ Therefore, due to |*J*_NNI_| ≫ |*J*_NNNI_|, the *J*_NNNI_ could always be virtually ferromagnetic although some frustration with *J*_NNNI_ < 0 (antiferromagnetic) likely occurred in the case of Fig. 6e. Interestingly, although the *l*_trans_ distance in 3⊃CO_2_ was longer than that in 3, the *T*_C_ of 3⊃CO_2_ shifted to a higher temperature. This implied that the sign of *J*_NNNI_ in 3 could be negative (antiferromagnetic); however, its much smaller value compared to *J*_NNI_ in competing contributions suggested that 3 was an F (Fig. 6e). Thus, the insertion of CO_2_ into 3 converted the antiferromagnetic *J*_NNNI_ in the original 3 into ferromagnetic *J*_NNNI_ (>0) in 3⊃CO_2_ (Fig. 6f), which produced an F of 3⊃CO_2_ with a higher *T*_C_ (Fig. 7b).

## Conclusions

An efficient and straightforward strategy for ON/OFF switching of the magnetic ground state (*i.e.*, magnetic phase) is to invert the sign of the inter-layer magnetic interaction in a layered magnet. The inter-layer magnetic interaction is commonly associated with the inter-layer distance and the stacking modes that define the inter-layer environment. In this study, we demonstrated that the magnetic phase changes were possible by *in situ* tuning of the inter-layer distance using common gases such as CO_2_, O_2_, and N_2_. In particular, 1 revealed a drastic magnetic phase change from an AF to F in 1⊃CO_2_ without a change in the charge-ordered state although 1⊃N_2_ and 1⊃O_2_ were still AFs but different from pristine 1. These results confirm that the magnetic phase can be tuned by the types of gas accommodated even when common gases are used.

All common-gas-responsive porous magnets reported so far have been limited to being “magnet erasing,” which includes a change from an F to AF upon gas adsorption.^43,46^ The present work especially offers the first example of a change from an AF to F depending on the type of gas. The sign of the inter-layer magnetic interaction (*J*_NNNI_) *vs.* the inter-layer distance (*l*_trans_ or *l*′_trans_) is in good agreement with the empirical rule for the selected series of [Ru_2_]_2_–TCNQ layered magnets.^32,44,64,65^ Therefore, it is concluded that the phase change phenomenon described in this work originates purely from structural modification. The key factor for this mechanism is the presence of anisotropic combinations in the magnetic interactions, such as strong intralayer spin couplings and weak inter-layer dipole interactions based on low-dimensional layered systems. The latter contribution is a variable parameter that enables tuning of not only the magnitude of the interaction but also the sign of the exchange required for bulk phase changes. In other words, gas molecules, even common gases, act as good tools to modify the inter-layer dipole interactions. Hence, not only the [Ru_2_]_2_–TCNQ systems but also other families of porous layered AFs with strong intralayer and weak inter-layer interactions could be promising candidates for magnetic switching *via* similar mechanisms.

## Data availability

All data supporting the findings of this study, including the details of the experimental study, are available in the article and ESI.†

## Author contributions

W. K. and H. M. conceived the study. H. N. and K. N. prepared and characterized the materials. W. K., H. N., and K. N. recorded the PXRD data at the laboratory level, and S. K. and K. S. recorded the PXRD data using a synchrotron X-ray beam at the Super Photon ring (Spring-8). W. K., H. N., and K. N. carried out the experiments of gas adsorption, *in situ* IR spectroscopy, and *in situ* gas adsorption–magnetic measurements. W. K. analyzed the structures of 1 and 1⊃gas using Rietveld refinement techniques by using synchrotron PXRD data. H. M. supervised all the experiments. W. K. and H. M. prepared the original draft, and all authors discussed the results and commented on the manuscript.

## Conflicts of interest

There are no conflicts to declare.

## Supplementary Material

SC-014-D2SC06337A-s001

SC-014-D2SC06337A-s002
