# Supplementary material for: Inter-layer magnetic tuning by gas adsorption in π-stacked pillared-layer framework magnets
Source: Chem Sci. 2022 Dec 26;14(4):791–800. doi: 10.1039/d2sc06337a (PMC9890543; doi:10.1039/d2sc06337a)
Supplement: SC-014-D2SC06337A-s001 [file SC-014-D2SC06337A-s001.pdf]

Electronic Supplementary Material (ESI) for Chemical Science.
This journal is © The Royal Society of Chemistry 2022

Inter-Layer Magnetic Tuning by Gas Adsorption in π-Stacked Pillared-Layer Framework Magnets

Wataru Kosaka,[a,b] Honoka Nemoto,[b] Kohei Nagano,[b] Shogo Kawaguchi,[c] Kunihisa Sugimoto,[c,d] Hitoshi Miyasaka*[a,b]

[a] Institute for Materials Research, Tohoku University, 2-1-1 Katahira, Aoba-ku, Sendai 980-8577, Japan

[b] Department of Chemistry, Graduate School of Science, Tohoku University, 6-3 Aramaki-Aza-Aoba, Aoba-ku, Sendai 980-8578, Japan

[c] Diffraction & Scattering Division Japan Synchrotron Radiation Research Institute (JASRI), 1-1-1 Kouto, Sayo-cho, Sayo-gun, Hyogo 679-5198, Japan

[d] Department of Chemistry, Kindai University, 3-4-1 Kowakae, Higashi-Osaka, Osaka 577-8502, Japan

E-mail: miyasaka@imr.tohoku.ac.jp; Tel: +81-22-215-2030; FAX: +81-22-215-2031

■ Contents for SI

Contents for SI S2

Experimental section S3

Table S1 Gas pressure dependence of lattice constants of **1** S6

Table S2 Crystallographic data obtained from Rietveld refinement for **1⊃GAS** S7

Table S3 Gas pressure dependence of lattice constants of **2** S8

Table S4 Gas pressure dependence of lattice constants of **3** S9

Fig. S1 PXRD patterns and Rietveld refinement for **1⊃GAS** S10

Fig. S2 IR spectra of **1** measured under gas S11

Fig. S3 Magnetic properties for **1⊃**$\mathbf{N_2}$ S12

Fig. S4 Magnetic properties for **1⊃**$\mathbf{O_2}$ S13

Fig. S5 *M*−*T* curves of **1⊃**$\mathbf{CO_2}$ S14

Fig. S6 Reversibility of alternation on magnetic properties upon gas adsorption for **1** S15

Fig. S7 Schematic representation of interlayer environment S16

Structural details of 2 and 3 .... S17

Fig. S8 PXRD patterns for **2⊃GAS** S18

Fig. S9 PXRD patterns for **3⊃GAS** S19

Fig. S10 PXRD patterns and LeBail analyses for **2⊃GAS** S20

Fig. S11 PXRD patterns and LeBail analyses for **3⊃GAS** S21

Fig. S12 IR spectra of **2** and **3** measured under gas S22

Fig. S13 *M*−*T* curves of **2⊃GAS** at 100 Oe S23

Fig. S14 *M*−*T* curves of **2⊃GAS** at 5 Oe S24

Fig. S15 *M*−*H* curves of **2⊃GAS** and **3⊃GAS** S25

Fig. S16 *M*−*T* curves of **3⊃GAS** at 100 Oe S26

Fig. S17 *M*−*T* curves of **3⊃GAS** at 5 Oe S27

References in SI S28

■ EXPERIMENTAL SECTION

General procedures and materials

All solvents were dried using common drying agents and distilled under nitrogen before use. All syntheses were performed in an inert atmosphere using standard Schlenk-line techniques in a commercial glove box under a nitrogen atmosphere. All chemicals were of reagent grade and were purchased from commercial sources. [MCp$^*_2$] (M = Co, Fe, and Cr) and TCNQ were purchased from Sigma-Aldrich Co., LLC. Starting material [Ru_2(2,3,5,6-F_4PhCO_2)$_4$(THF)$_2$] was prepared according to a previously reported method.[1] The synthesis of [MCp$^*_2$][{$Ru_2^{II,II}$(2,3,5,6-F_4PhCO_2)$_4$}$_2$(TCNQ)] (M = Co, Fe, and Cr) has been previously described.[2]

Physical measurement

PXRD patterns were collected in the laboratory using a Rigaku Ultima IV diffractometer with Cu $K\alpha$ radiation (λ = 1.5418 Å). The ground samples were sealed in a soda-lime glass capillary with an inner diameter of 0.5 mm, and the PXRD patterns were obtained with 0.02° steps. Magnetic susceptibility measurements were conducted using a SQUID magnetometer (Quantum Design MPMS-XL) within 1.8–300 K and −7–7 T. The diamagnetic contribution was removed from the experimental data using Pascal constants.[3]

Crystallography based on Rietveld refinement of PXRD data

A ground sample of **1** was sealed in a silica glass capillary with an inner diameter of 0.4 mm. The PXRD data for the structural analyses of **1**$\supset$**CO**$_2$, **1**$\supset$**O**$_2$, and **1**$\supset$**N**$_2$ were collected using a synchrotron X-ray beam and a diffractometer with a multimodular system constructed using six MYTHEN detectors on the BL02B2 beamline at the Super Photon ring (SPring-8).[4] The measurement temperature was controlled by flowing low-temperature nitrogen gas. The gas-handling system consisting of valves and pressure gauges for gas dosing and removing was connected to the goniometer head using a stainless steel line to obtain *in situ* PXRD patterns. Cell parameters were determined using the DIFFRACplus TOPAS® v4.2 software. Crystal structure of **2** obtained by single crystal X-ray diffraction analysis was used to construct the initial structural model.[1] Next, structural refinement using the Rietveld method with the RIETAN-FP program was applied.[5] The peak shape was modeled using the split-Pearson VII function. Soft constraints on the bond angles and bond distances were adapted throughout the refinement. After the framework structure was refined, gas molecules were located using the FOX software[6] with a parallel tempering algorithm while the position of the framework was fixed. Subsequently, the entire structure was refined. In the initial refinement stage, hydrogen atoms were removed from the structural model. After all parameters were refined, hydrogen atoms were attached to the calculated positions, followed by refinement of all parameters. The fractional coordinates of hydrogen atoms were not initially refined. After refinement, the fractional coordinates of the hydrogen atoms were again calculated and modified. This process was repeated until the fractional coordinates of hydrogen atoms became self-consistent. The crystallographic data and

Rietveld refinement results are summarized in Table S2. The CIF data for **1⊃CO_2**, **1⊃O_2**, and **1⊃N_2** were deposited at the Cambridge Crystallographic Data Centre (CCDC) in Supplementary Publication No. CCDC–2219754, –2219755, and –2219756 for **1⊃CO_2**, **1⊃O_2**, and **1⊃N_2**, respectively. Copies of data can be obtained free of charge from the CCDC via https://www.ccdc.cam.ac.uk/structures/. The solvent-accessible volume was estimated using the PRATON software.[7] Structural diagrams were prepared using the VESTA software.[8] The Connolly surface was depicted with a probe radius of 1.4 Å using the Discovery Studio Visualizer software.[9]

Gas adsorption measurements

Adsorption isotherm measurements for N_2 (at 120 K), O_2 (at 90 and 120 K), and CO_2 (at 195 K) were performed using an automatic volumetric adsorption apparatus (BELSORP MAX, Microtrac BEL) connected to a cryostat system (ULVAC-Cryo). A known weight (~30 mg) of the dried sample was placed into the sample cell. Next, before measurements, the cell was evacuated using the degassing function of the analyzer for 12 h at 353 K. The change in pressure was monitored, and the amount of adsorbed gas was determined by the decrease in pressure at the equilibrium state. When the reading of the pressure gauge for 500 seconds was within 0.3%, the system was judged to have reached adsorption equilibrium.

In situ infrared spectroscopy

In situ infrared spectroscopy was conducted through CaF_2 windows on a cryostat system (RC102, CRYO Industries) connected to a gas-handling and pressure monitoring system (BELSORP MAX, Microtrac BEL) with a transmission configuration using a JASCO FT-IR 4200 spectrometer. The neat samples were sandwiched between two CaF_2 plates.

In situ magnetic measurements under gases

The polycrystalline samples were placed in gelatin capsules. A piece of cotton was placed above the sample to prevent sample movement during gas adsorption. The capsule was then held at the center of the plastic straw. The straw was attached to the edge of a homemade sample rod made of a stainless steel tube and a brass male thread with fluorocarbon tape.[10] The sample was isolated from the surrounding atmosphere by overlaying a closed-ended brass tube, which could be attached to the thread on the open end of the sample rod using screws. An airtight seal between the thread and the brass tube was achieved using a silicon sealant (CAF® 4, Bluestar Silicones). The stainless steel tube was connected to a gas-handling system with a turbomolecular pump and manometer (BELSORP MAX, Microtrac BEL). Subtracting the background signal from the brass tube was unnecessary.

In situ PXRD at laboratory

The ground sample was sealed in a soda-lime glass capillary with an inner diameter of 0.5 mm. The PXRD pattern was obtained with 0.02° steps using a Rigaku Ultima IV diffractometer with Cu Kα radiation (λ = 1.5418 Å). To obtain the PXRD patterns under gas-adsorbed conditions, the glass capillary was connected to stainless steel lines that possessed valves to dose and remove gas, which were connected to a gas-handling system (BELSORP MAX, MicrotracBEL). The temperature was controlled using a stream of N_2 gas. A Le Bail profile fitting algorithm, which affords refined cell parameters, was applied using the RIETAN-FP program.[5]

Table S1 Gas pressure dependence of lattice constants of **1** determined by PXRD measurements and Le Bail whole patter decomposition or Rietveld method.

T (K)	P (kPa)	a (Å)	b (Å)	c (Å)	α (deg)	β (deg)	γ (deg)	V (Å^3)	l_V (Å) [a]	R_{wp} [b]
@CO_2										
195	0 (vac)	10.4167(4)	14.2814(5)	16.1694(8)	97.088(4)	108.944(4)	99.410(4)	2204.03(17)	9.618	2.500
	1	10.4110(5)	14.3140(6)	16.1706(9)	97.244(5)	108.649(5)	99.654(5)	2208.9(2)	9.620	2.521
	3	10.4421(5)	14.3517(7)	16.1966(9)	97.391(5)	108.542(5)	99.786(6)	2224.0(2)	9.648	2.608
	10	10.5459(6)	14.3555(7)	16.1957(10)	97.989(6)	108.800(5)	99.354(6)	2241.6(2)	9.736	2.351
	15	10.6414(5)	14.3494(6)	16.2318(8)	98.417(5)	109.248(5)	98.752(6)	2260.9(2)	9.812	2.355
	20	10.6891(4)	14.3452(5)	16.2534(7)	98.631(4)	109.531(3)	98.422(4)	2269.95(17)	9.847	2.495
	30	10.7421(4)	14.3479(5)	16.2686(7)	98.757(4)	109.786(3)	98.239(3)	2280.14(16)	9.884	2.679
	40	10.7622(3)	14.3454(4)	16.2749(6)	98.819(3)	109.862(3)	98.128(3)	2284.09(14)	9.900	2.508
	60	10.7897(3)	14.3491(4)	16.2805(6)	98.914(3)	109.969(3)	98.024(3)	2289.58(14)	9.921	2.640
	100 [c]	10.8295(4)	14.3466(5)	16.2889(7)	98.991(3)	110.110(4)	97.830(4)	2297.51(17)	9.954	3.480
@O_2										
	0 (vac) [c,d]	10.3802(6)	14.2317(7)	16.1613(12)	96.885(4)	109.144(5)	99.487(5)	2185.5(3)	9.571	2.470
120	100 [c]	10.3561(6)	14.2275(7)	16.1588(10)	96.889(4)	109.083(6)	99.555(5)	2179.7(2)	9.550	6.490
@N_2										
120	100 [c]	10.3569(5)	14.2210(3)	16.1573(9)	96.891(3)	109.135(5)	99.504(5)	2178.3(2)	9.549	2.444

[a] Inter-layer vertical distance (Figure S7). [b] $R_{wp} = [\sum w[y - f(\boldsymbol{x})]^2/\sum wy^2]^{1/2}$, where y and $f(\boldsymbol{x})$ represent the observed intensity and the calculated intensity at a diffraction angle of 2θ, respectively. [c] Refined by Rietveld method. [d] Ref. 2.

Table S2 Crystallographic data obtained from Rietveld refinement for **1-GAS**.

	**1⊃CO_2**	**1⊃O_2**	**1⊃N_2**
Formula	$C_{92}H_{42}CoF_{32}N_4O_{24}Ru_4$	$C_{88}H_{42}CoF_{32}N_4O_{20}Ru_4$	$C_{88}H_{42}CoF_{32}N_6O_{16}Ru_4$
formula weight	2658.49	2546.45	2510.47
crystal system	Triclinic	Triclinic	Triclinic
space group	$P-1$	$P-1$	$P-1$
a / Å	10.8295(4)	10.3561(6)	10.3569(5)
b / Å	14.3466(5)	14.2275(7)	14.2210(3)
c / Å	16.2889(7)	16.1588(10)	16.1573(9)
α / deg	98.991(3)	96.889(4)	96.891(3)
β / deg	110.110(4)	109.083(6)	109.135(5)
γ / deg	97.830(4)	99.555(5)	99.504(5)
V / Å^3	2297.50(17)	2179.7(2)	2178.3(2)
Z	1	1	1
T / K	195(1)	120(1)	120(1)
D_{calc} / g·cm^{-3}	1.921	1.940	1.914
F_{000}	1305.0	1249.0	1231.0
λ / Å	0.799690(1)	0.799690(1)	0.799690(1)
$2\theta_{max}$ / deg	39.996	39.996	39.996
$2\theta_{min}$ / deg	2.100	2.100	2.100
step size / deg	0.006	0.006	0.006
R_{wp} [a]	0.03480	0.02545	0.02444
R_B [b]	0.01986	0.01916	0.02258
CCDC No.	2219754	2219755	2219756

[a] $R_{wp} = [\sum w[y - f(\boldsymbol{x})]^2/\sum wy^2]^{1/2}$, where y and $f(\boldsymbol{x})$ represent the observed intensity and the calculated intensity at a diffraction angle of 2θ, respectively. [b] $R_B = \sum||F_o| - |F_c||/\sum|F_o|$.

Table S3 Gas pressure dependence of lattice constants of **2** determined by PXRD measurements and Le Bail whole patter decomposition.

T (K)	P (kPa)	a (Å)	b (Å)	c (Å)	α (deg)	β (deg)	γ (deg)	V (Å^3)	l_V (Å)[a]	R_{wp}[b]
@CO_2										
195	0 (vac)[c]	10.4347(6)	14.3690(7)	16.2094(10)	96.653(5)	108.398(6)	100.345(6)	2229.6(3)	9.637	4.195
	100	10.8718(11)	14.4140(11)	16.3595(17)	98.852(7)	109.958(8)	98.323(8)	2326.6(4)	9.986	4.974
@O_2										
	0 (vac)[c]	10.3817(12)	14.3777(13)	16.1938(19)	96.755(8)	108.024(10)	100.891(9)	2216.7(5)	9.571	2.470
120	100	10.4426(8)	14.4420(9)	16.2150(13)	97.046(5)	107.916(7)	101.147(6)	2239.1(3)	9.634	3.850
@N_2										
120	100	10.3585(11)	14.3455(12)	16.1895(18)	96.659(7)	108.151(10)	100.708(9)	2206.9(4)	9.567	5.284

[a] Inter-layer vertical distance (Figure S7). [b] $R_{wp} = [\sum w[y - f(\boldsymbol{x})]^2/\sum wy^2]^{1/2}$, where y and $f(\boldsymbol{x})$ represent the observed intensity and the calculated intensity at a diffraction angle of 2θ, respectively. [c] Ref. 2.

Table S4 Gas pressure dependence of lattice constants of **3** determined by PXRD measurements and Le Bail whole patter decomposition.

T (K)	P (kPa)	a (Å)	b (Å)	c (Å)	α (deg)	β (deg)	γ (deg)	V (Å^3)	l_V (Å) [a]	R_{wp} [b]
@CO_2										
195	0 (vac) [c]	10.6918(2)	14.3369(19)	16.1652(3)	97.964(12)	109.278(14)	99.029(16)	2261.3(7)	9.852	3.844
	100	10.9336(17)	14.4119(17)	16.304(3)	99.033(11)	109.589(16)	98.202(14)	2337.2(7)	10.071	7.571
@O_2										
	0 (vac) [c]	10.5456(2)	14.3005(2)	16.1316(3)	97.594(14)	109.003(17)	99.640(17)	2221.8(8)	9.716	3.446
120	100	10.609(2)	14.356(3)	16.163(4)	97.767(16)	108.859(19)	99.47(2)	2250.6(9)	9.789	6.490
@N_2										
120	100	10.613(2)	14.392(3)	16.203(3)	97.737(15)	108.617(18)	99.864(19)	2262.8(9)	9..792	6.434

[a] Inter-layer vertical distance (Figure S7). [b] $R_{wp} = [\sum w[y - f(\boldsymbol{x})]^2/\sum wy^2]^{1/2}$, where y and $f(\boldsymbol{x})$ represent the observed intensity and the calculated intensity at a diffraction angle of 2θ, respectively. [c] Ref. 2.

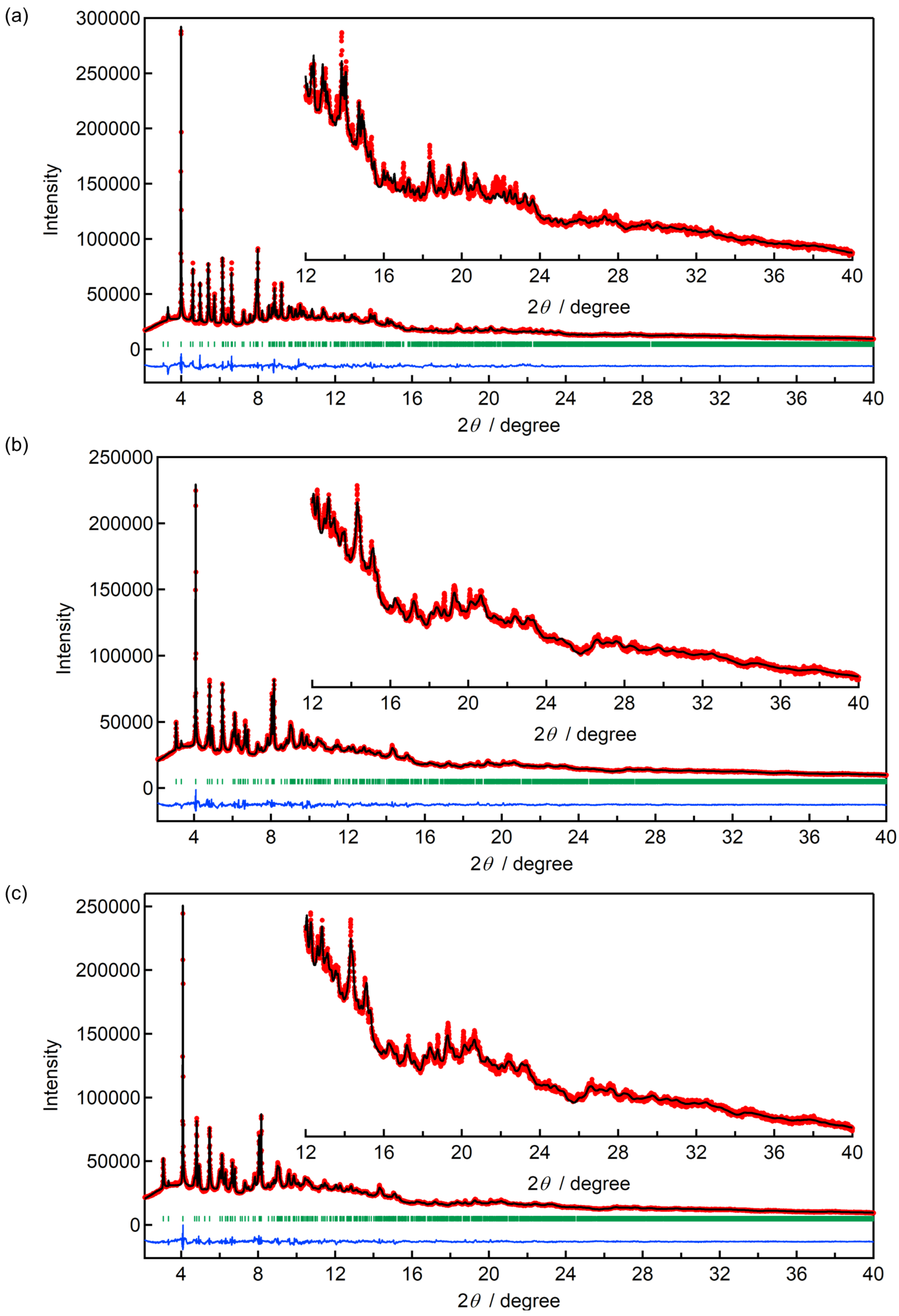

Fig. S1. PXRD pattern (λ = 0.80 Å) and Rietveld analysis for **1⊃CO_2** (a), **1⊃O_2** (b), and **1⊃N_2** (c), at 195 K, 120 K, and 120K, respectively. Red dots, black lines, and blue lines are the experimental plots, calculated pattern, and their difference, respectively. Green bars represent the calculated positions of the Bragg reflections.

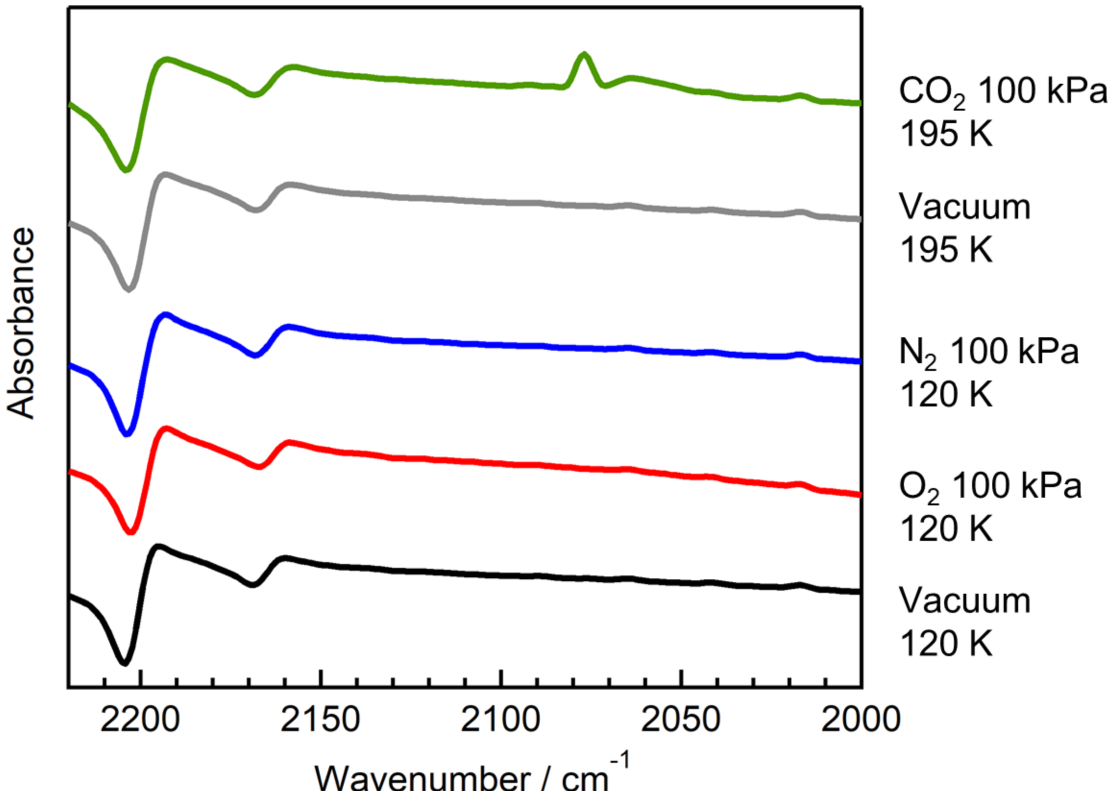

Fig. S2. IR spectra of **1** measured under 100 kPa of CO_2 at 195 K (green), O_2 at 120 K (red), N_2 at 120 K (blue), and vacuum at 195 K (gray) and 120 K (black).

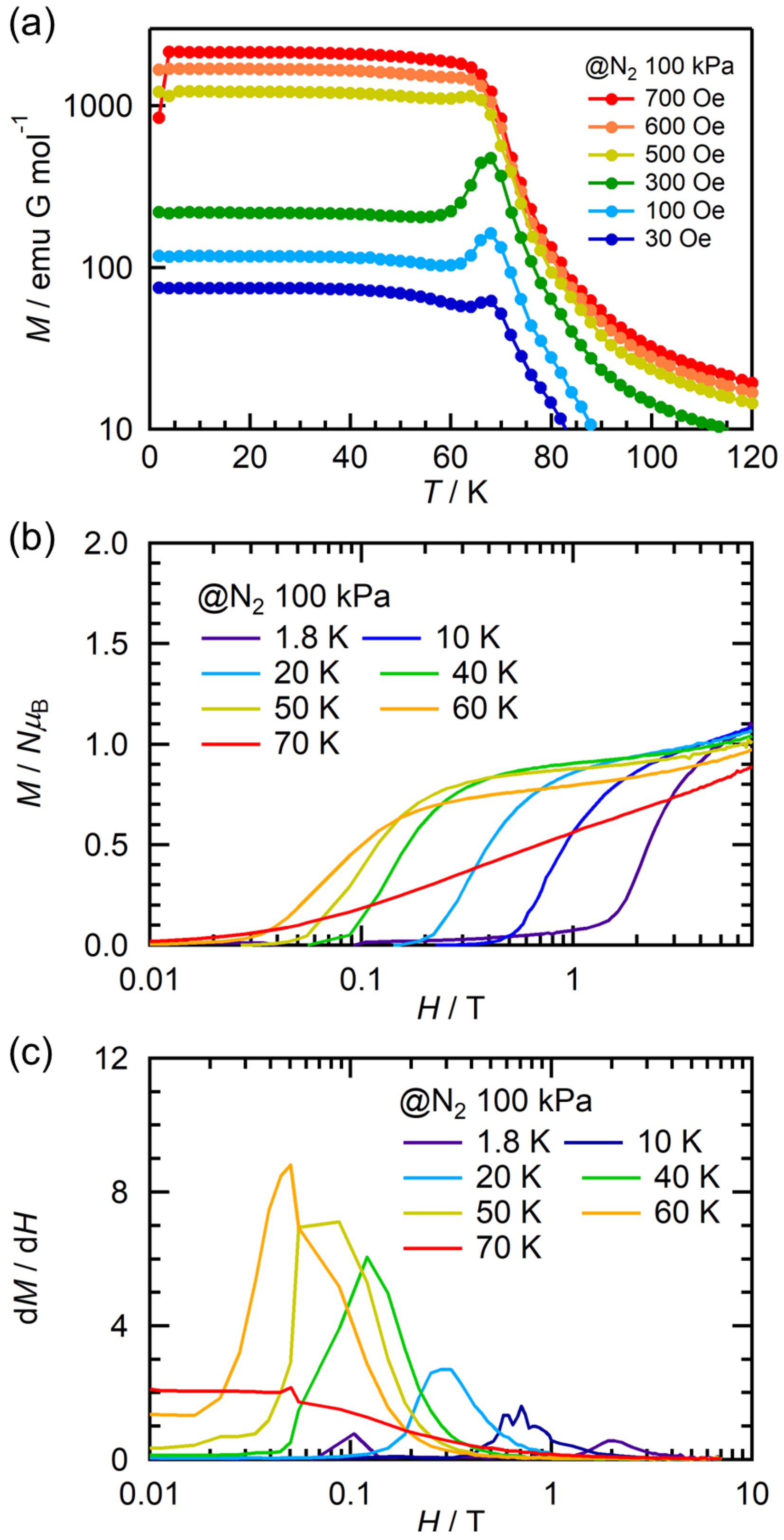

Fig. S3. Magnetic properties for **1⊃N₂** (P_{N2} = 100 kPa). FCM curves measured under different external fields (a), initial magnetization curves at several temperatures (b), and d*M*/d*H* plots for the initial magnetization at several temperatures (c).

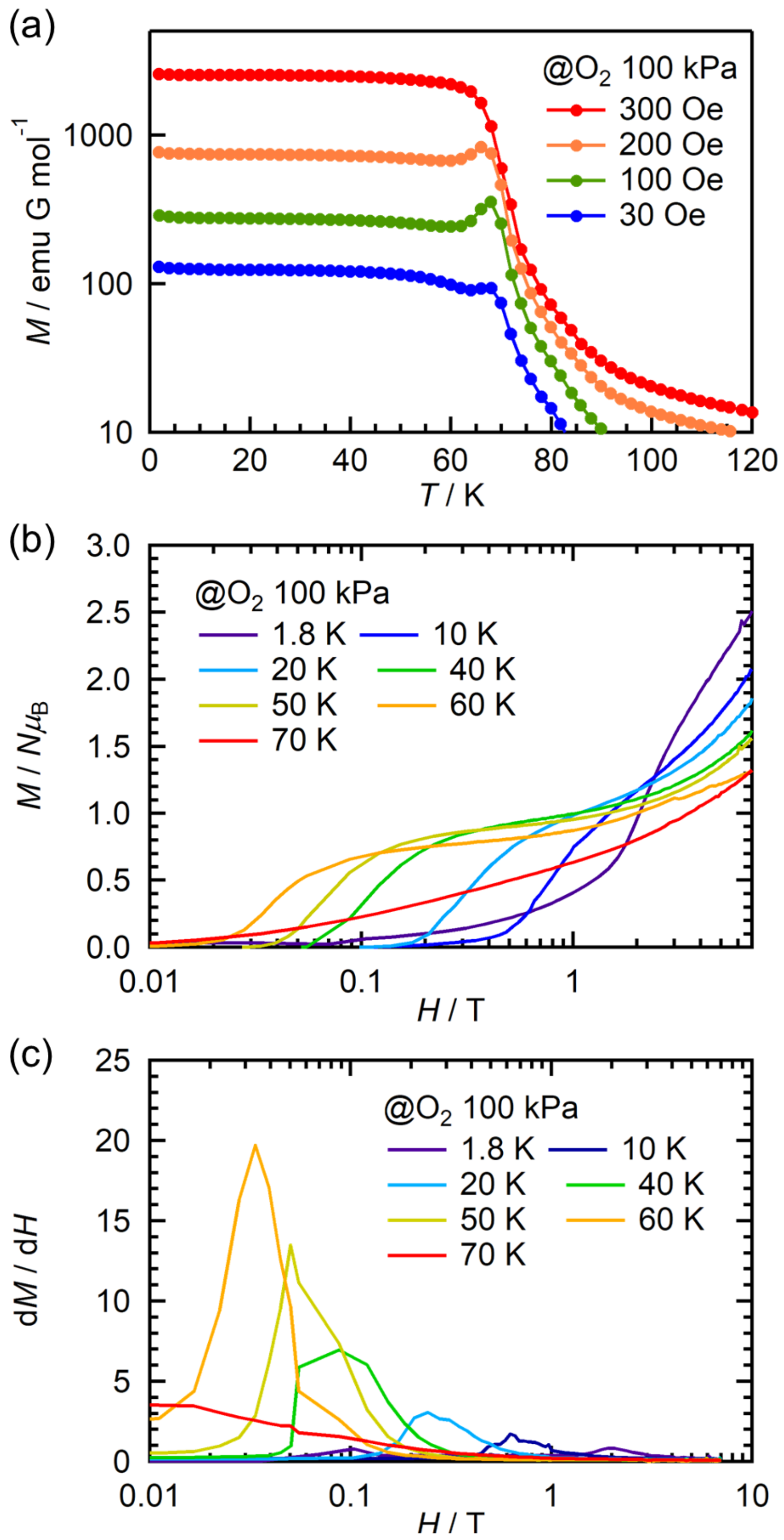

Fig. S4. Magnetic properties for **1⊃O₂** (P_{O2} = 100 kPa). FCM curves measured under different external fields (a), initial magnetization curves at several temperatures (b), and d*M*/d*H* plots for the initial magnetization at several temperatures (c).

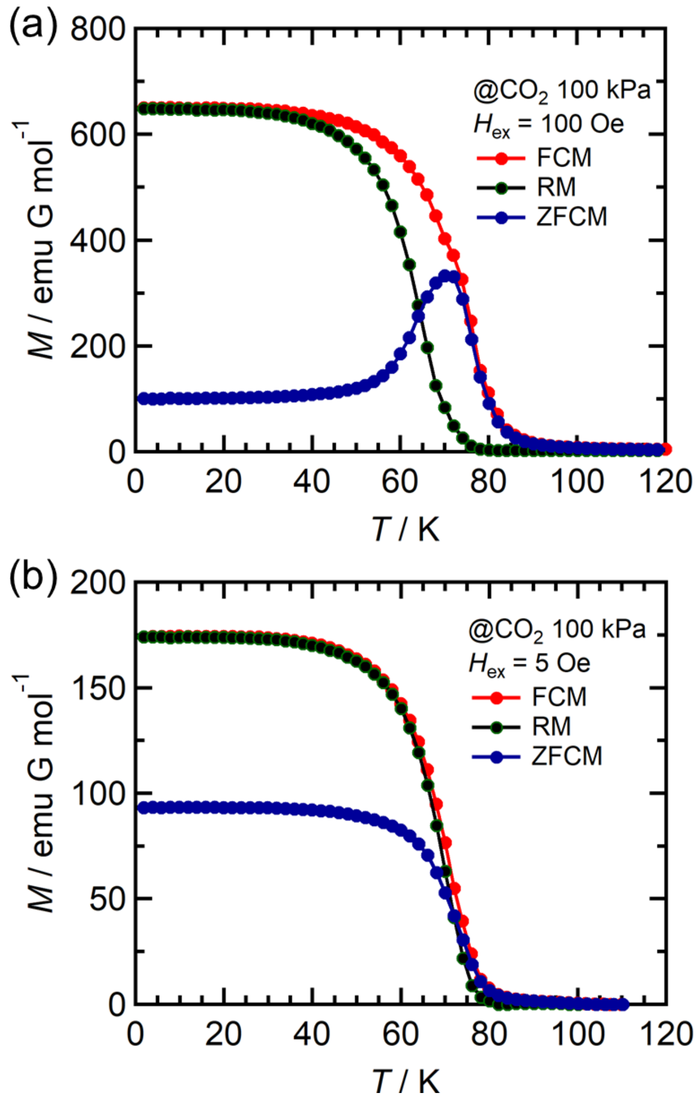

Fig. S5. FCM (red), RM (green), and zero-field-cooled magnetization (ZFCM, blue) curves for **1⊃CO₂** at 100 Oe (a) and 5 Oe (b).

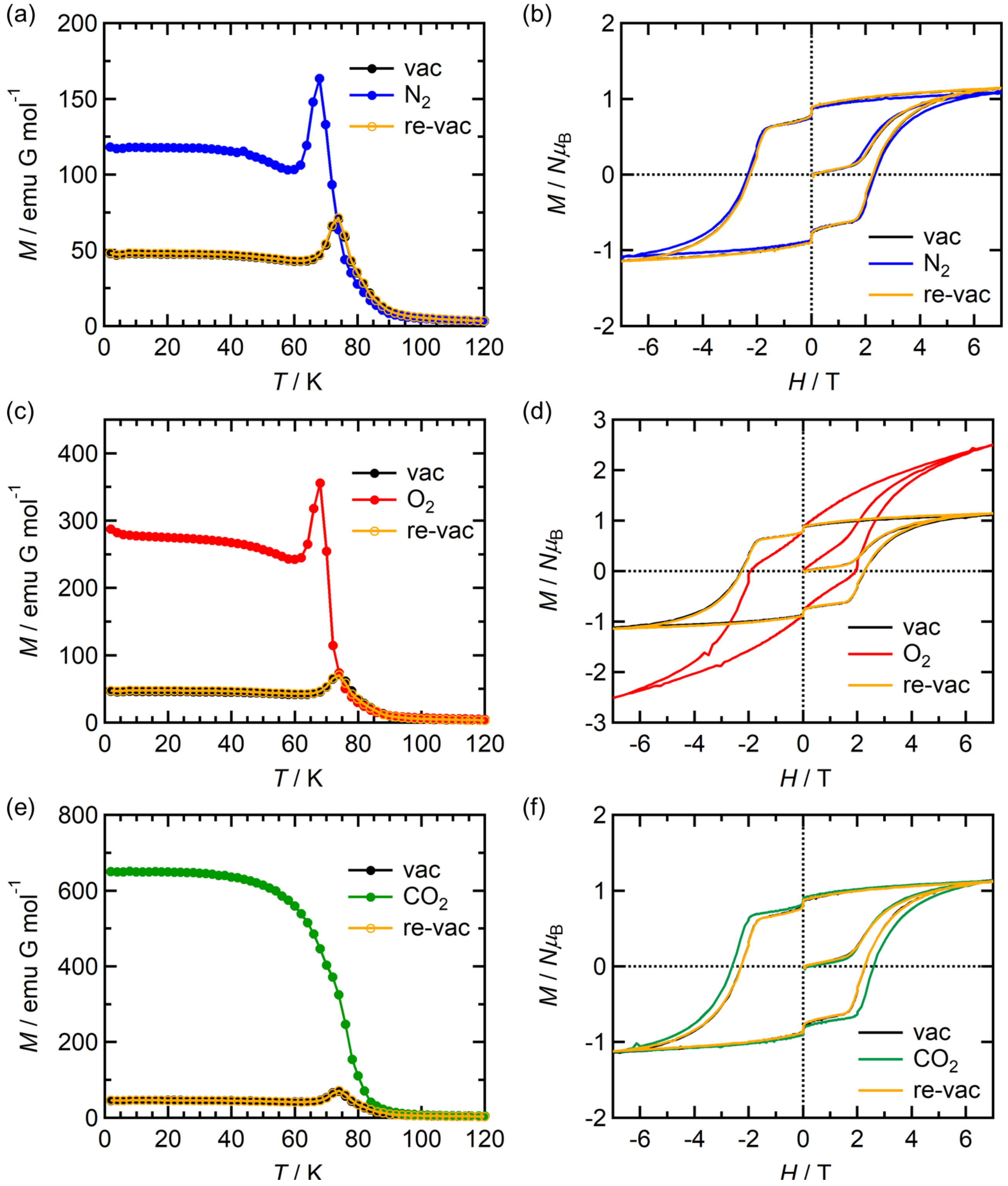

Fig. S6. FCM curves at 100 Oe (a, b, e) and *M*−*H* curves at 1.8 K (b, d, f) for **1** before gas introduction (black), anfter gas introduction (i.e., **1⊃GAS**; blue for N_2, red for O_2, and green for CO_2), and after gas desorption (yellow); N_2 (a, b), O_2 (c, d) and CO_2 (e, f).

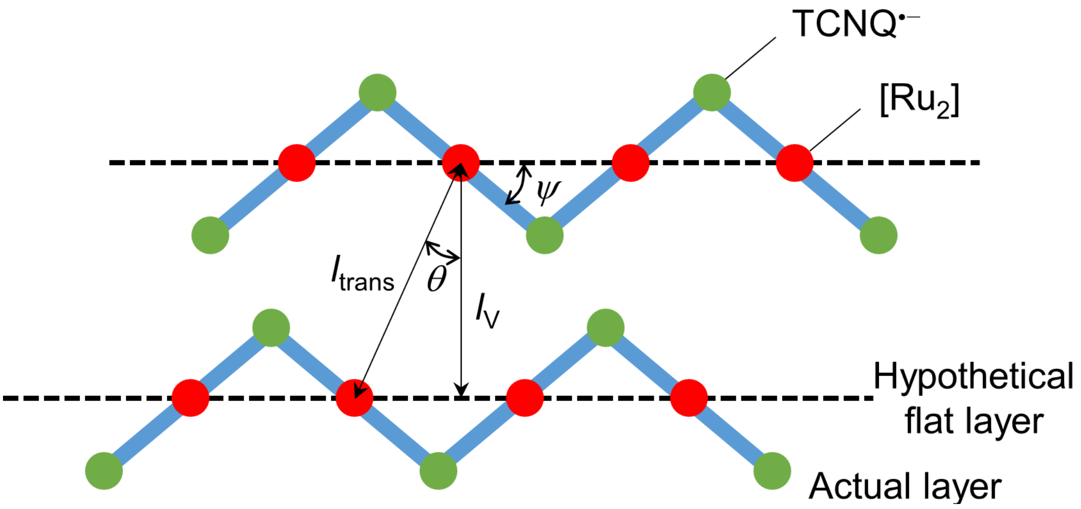

Fig. S7. Schematic representation of interlayer environment in the family of $[Ru_2]/TCNQR_x$ D_2A magnetic layered MOFs. $l_V = l_{trans} \cos \theta$.

Crystal structure under gases of 2 and 3 in SI

In situ PXRD was conducted to gain insights into the crystal structures under gas adsorption conditions. The PXRD patterns of **2** and **3** collected under vacuum and at 100 kPa of CO_2 at 195 K, and under vacuum and at 100 kPa of O_2 and N_2 at 120 K are shown in Fig. S8 and S9, respectively. The results of the Le Bail analysis of the PXRD patterns under gas adsorption are shown in Fig. S10 and S11, and the lattice constants of **2⊃gas** and **3⊃gas** obtained by Le Bail analysis are summarized in Tables S3 and S4, respectively. Similar to that of **1**, the inter-layer distance (l_{trans}) changed significantly before and after CO_2 adsorption; l_{trans} of **2** and **3** increased from 10.43 and 10.69 Å before CO_2 introduction to 10.87 and 10.93 Å under 100 kPa of CO_2 (+4% and +2%), respectively. The change in l_{trans} before and after O_2 or N_2 adsorption was small; for 100 kPa of O_2 (N_2), l_{trans} of **2** and **3** increased from 10.38 and 10.55 Å before O_2 (N_2) introduction to 10.44 (10.36) and 10.61 (10.61) Å for 100 kPa of O_2 (N_2), respectively.

The IR spectra of **2** and **3** were recorded at 100 kPa of CO_2, O_2, and N_2. The position of peak corresponding to the CN stretching mode in **2/3⊃CO_2**, **2/3⊃O_2**, and **2/3⊃N_2** remained unchanged in relation to that in **2/3** (Fig. S12), indicating no change in the electronic state before and after gas adsorption for any of the gases.

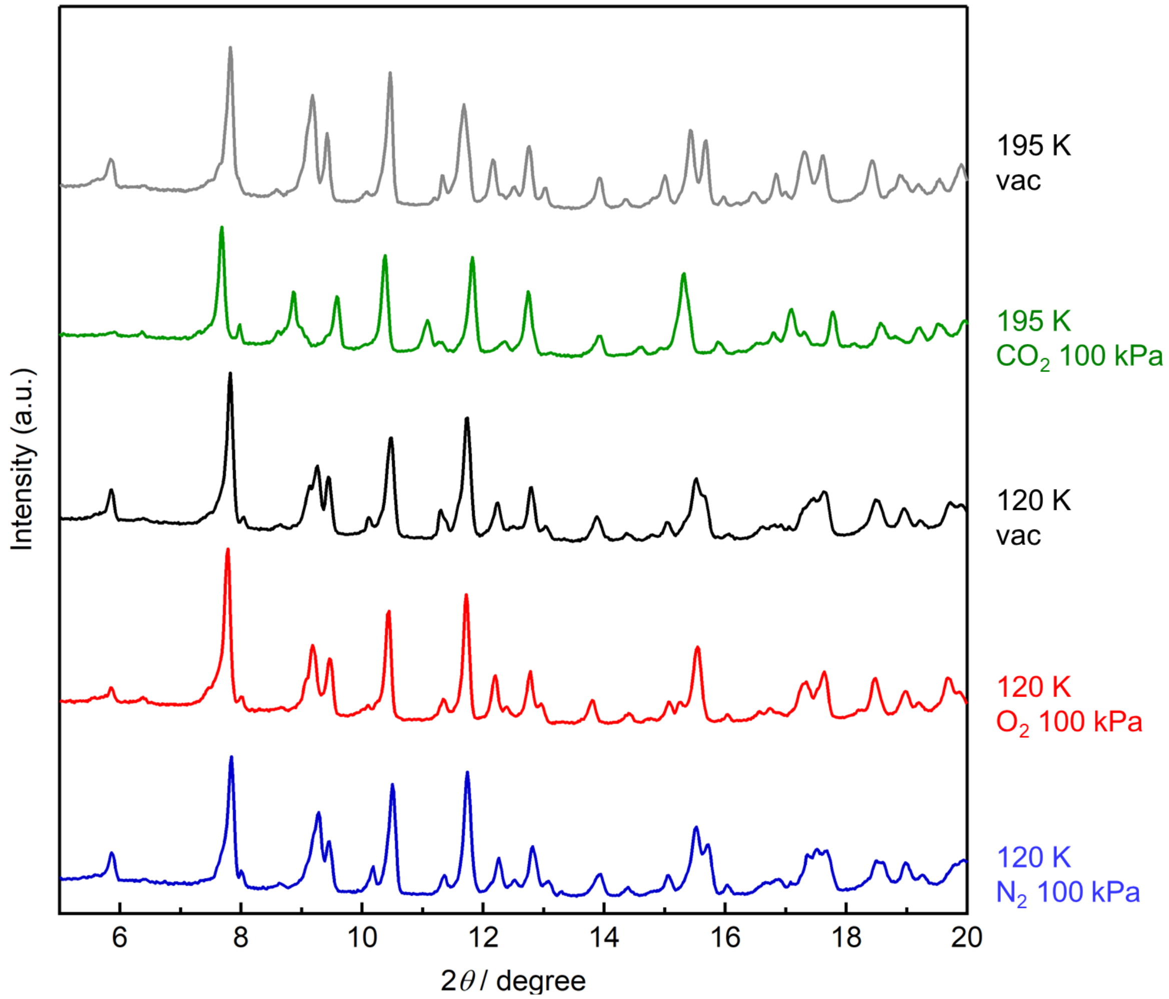

Fig. S8. The PXRD patterns (λ = 1.54 Å) of **2** measured at 195 K under vacuum (gray) and 100 kPa of CO_2 (green), and 120 K under vacuum (black) and 100 kPa of O_2 (red) and N_2 (blue).

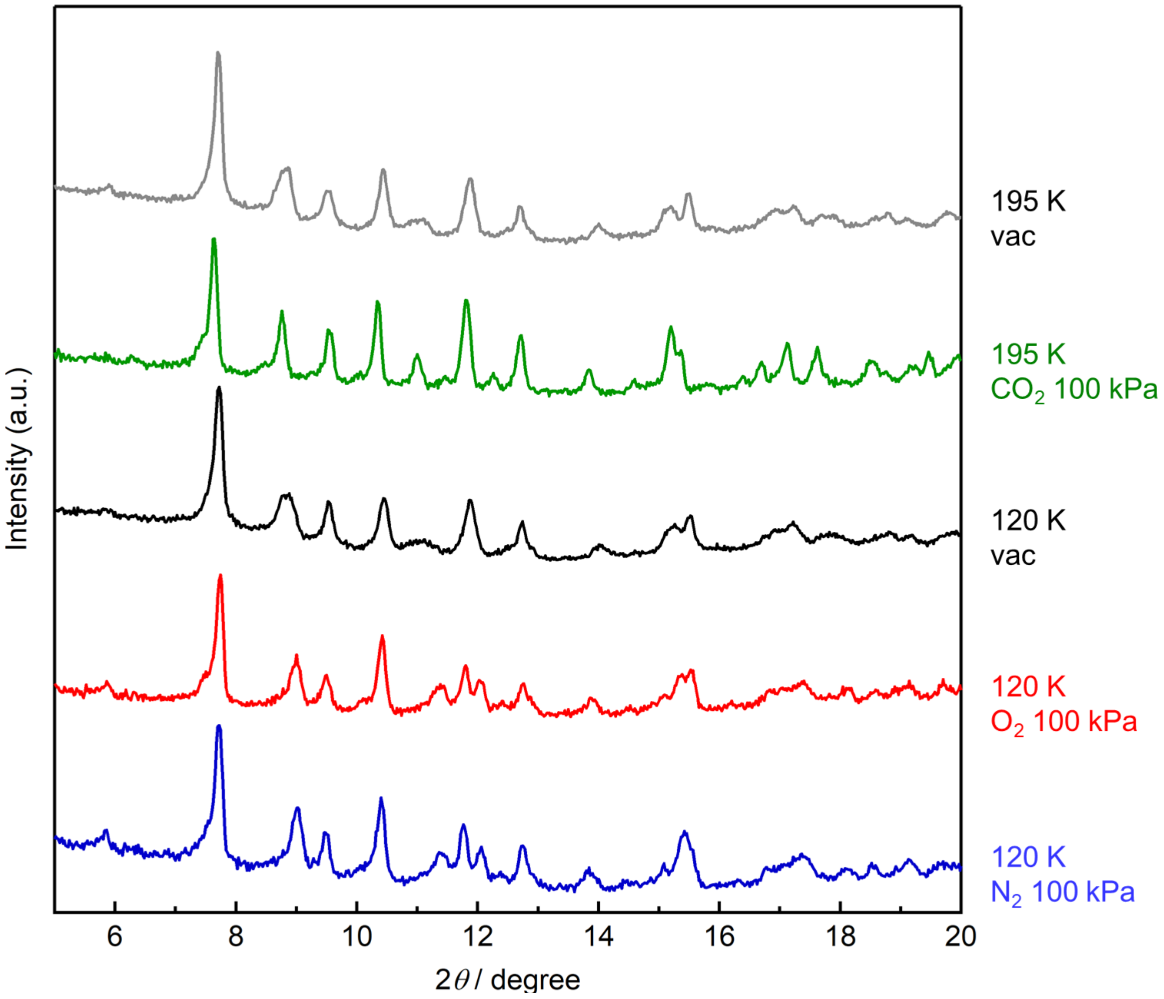

Fig. S9. The PXRD patterns (λ = 1.54 Å) of **3** measured at 195 K under vacuum (gray) and 100 kPa of CO_2 (green), and 120 K under vacuum (black) and 100 kPa of O_2 (red) and N_2 (blue).

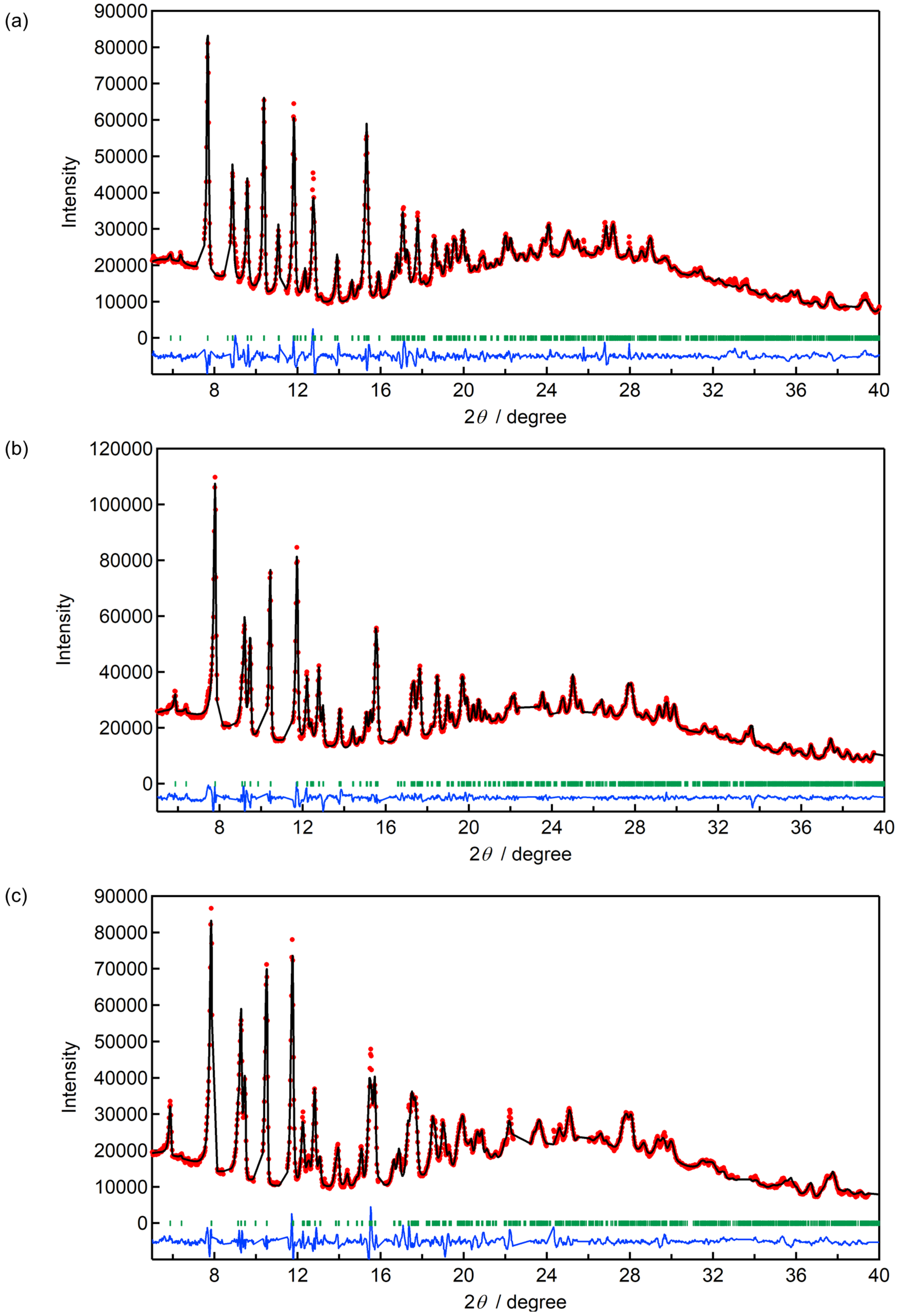

Fig. S10. PXRD pattern (λ = 1.54 Å) and Le Bail analysis for **2⊃CO₂** (a), **2⊃O₂** (b), and **2⊃N₂** (c), at 195 K, 120 K, and 120K, respectively. Red dots, black lines, and blue lines are the experimental plots, calculated pattern, and their difference, respectively. Green bars represent the calculated positions of the Bragg reflections.

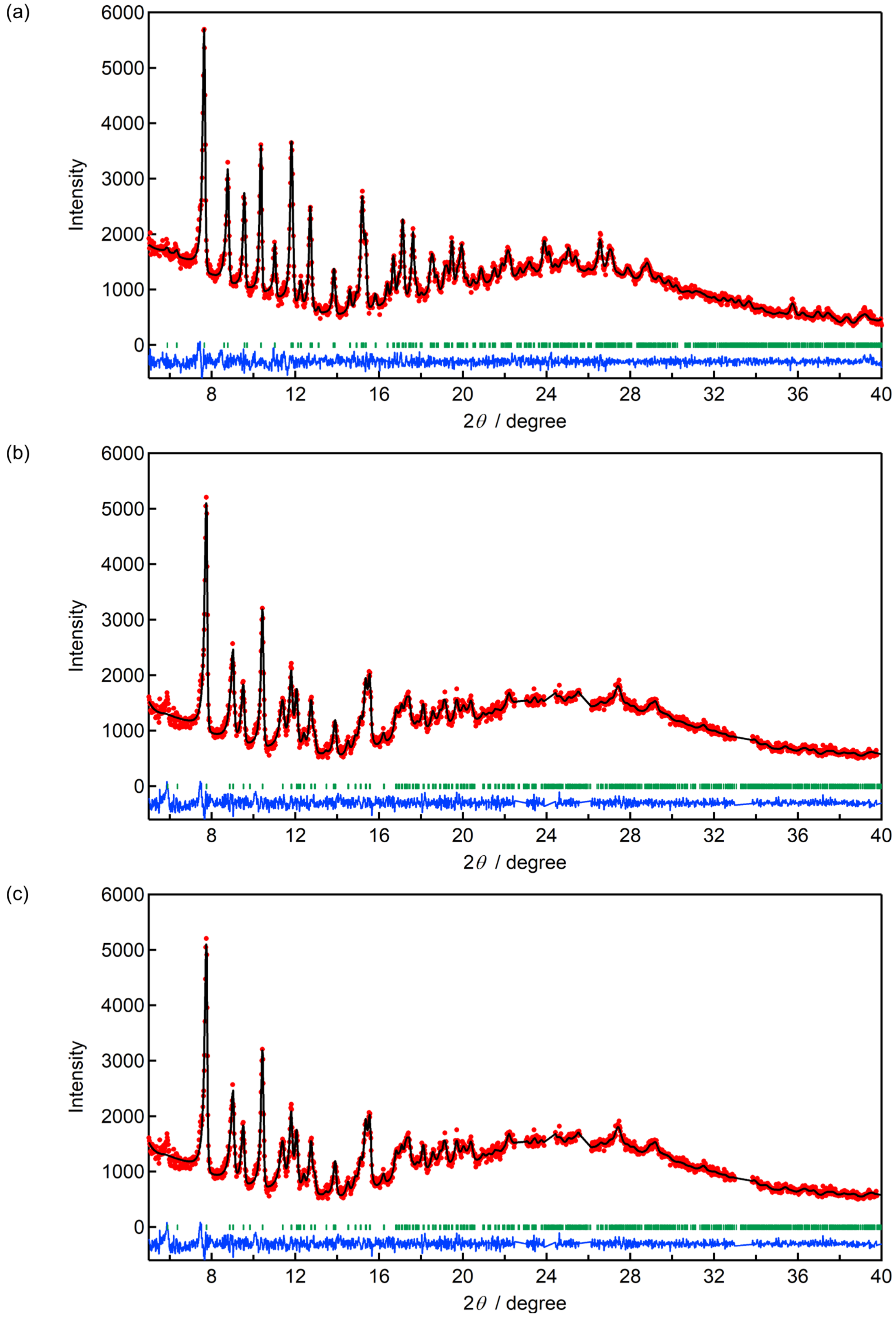

Fig. S11. PXRD pattern (λ = 1.54 Å) and Le Bail analysis for **3⊃CO₂** (a), **3⊃O₂** (b), and **3⊃N₂** (c), at 195 K, 120 K, and 120K, respectively. Red dots, black lines, and blue lines are the experimental plots, calculated pattern, and their difference, respectively. Green bars represent the calculated positions of the Bragg reflections.

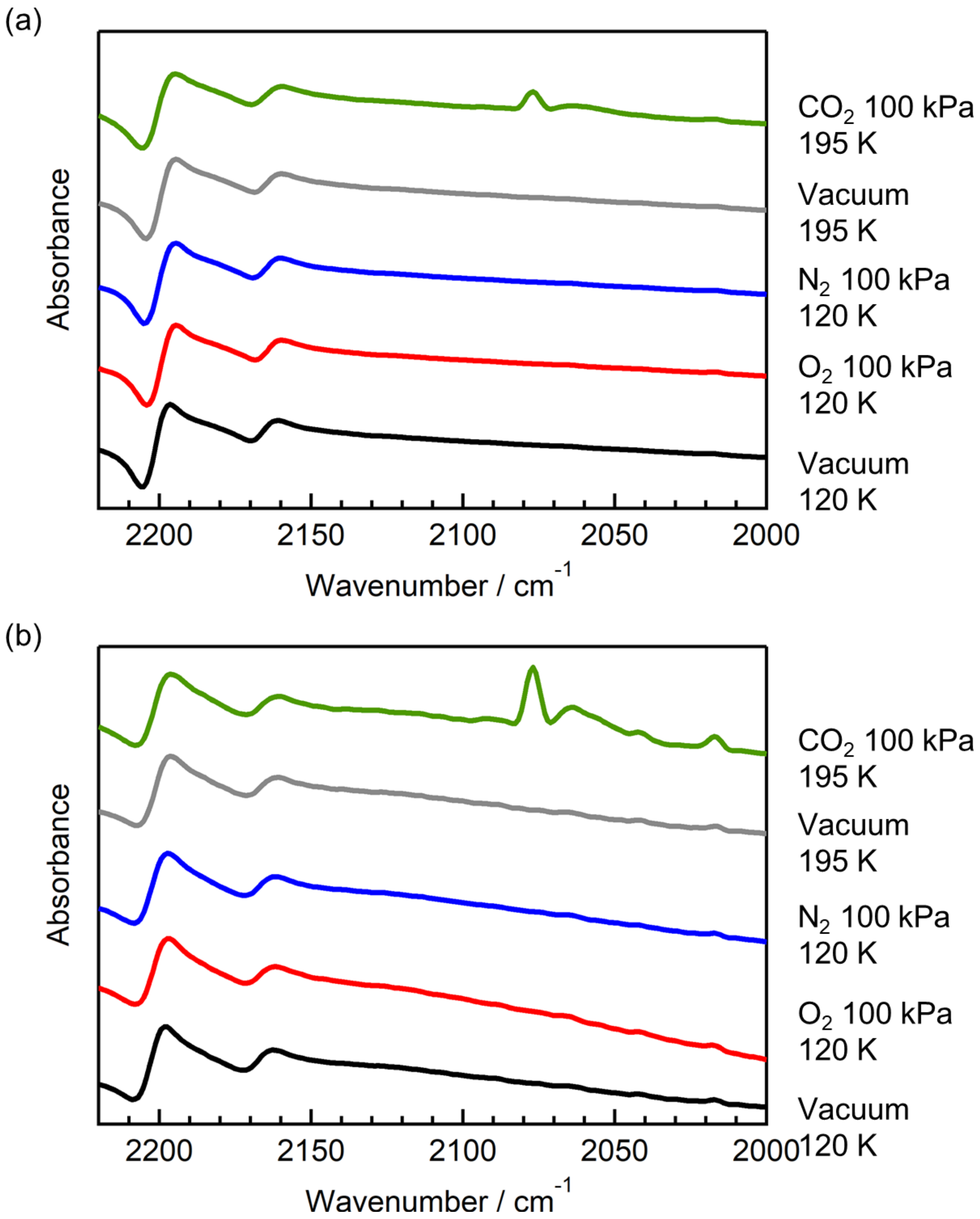

Fig. S12. IR spectra of **2** (a) and **3** (b) measured under 100 kPa of CO_2 at 195 K (green), O_2 at 120 K (red), N_2 at 120 K (blue), and vacuum at 195 K (gray) and 120 K (black).

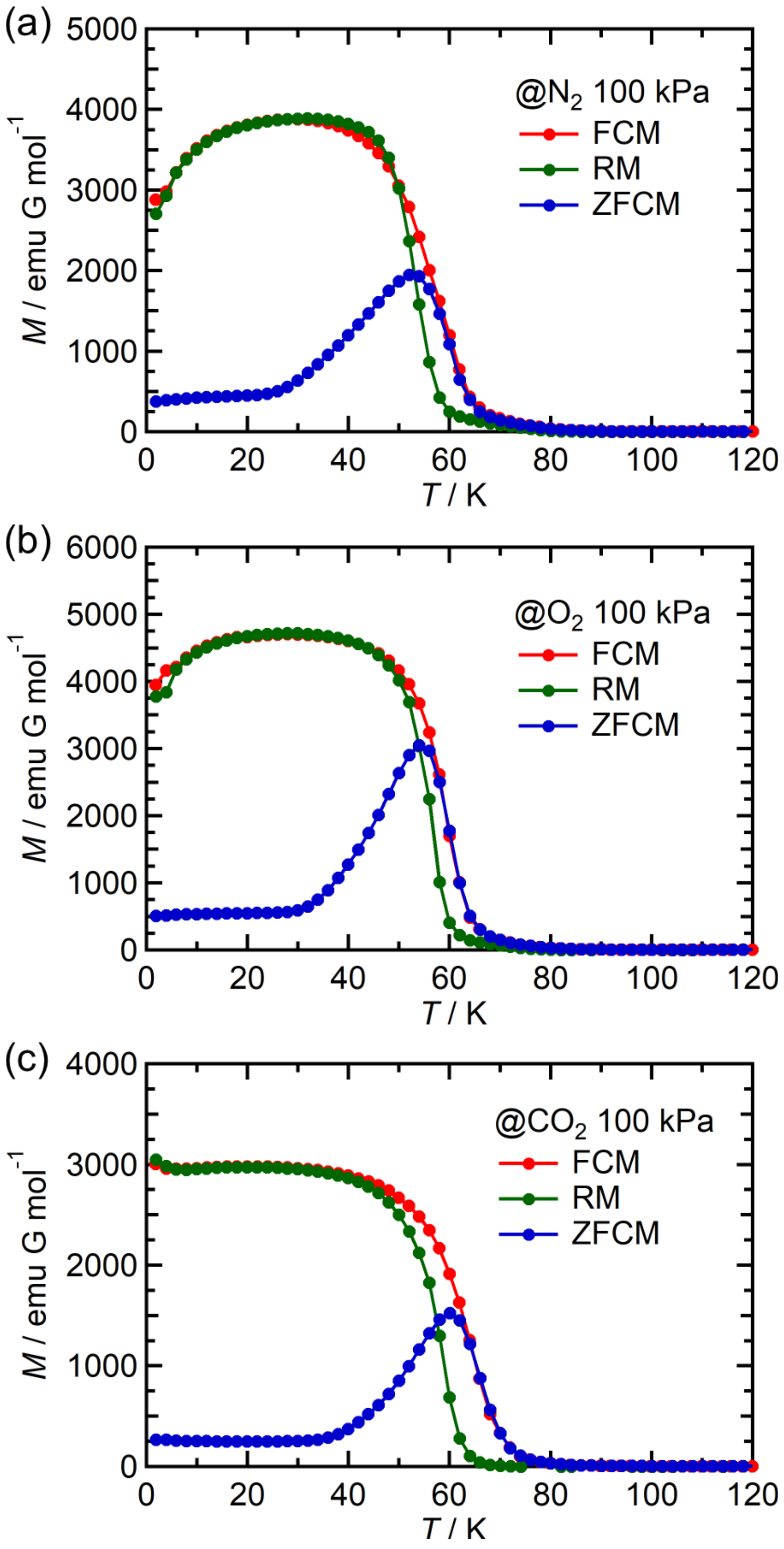

Fig. S13. FCM (red), RM (green), and zero-field-cooled magnetization (ZFCM, blue) curves at 100 Oe for **2⊃N_2** (a), **2⊃O_2** (b), and **2⊃CO_2** (c).

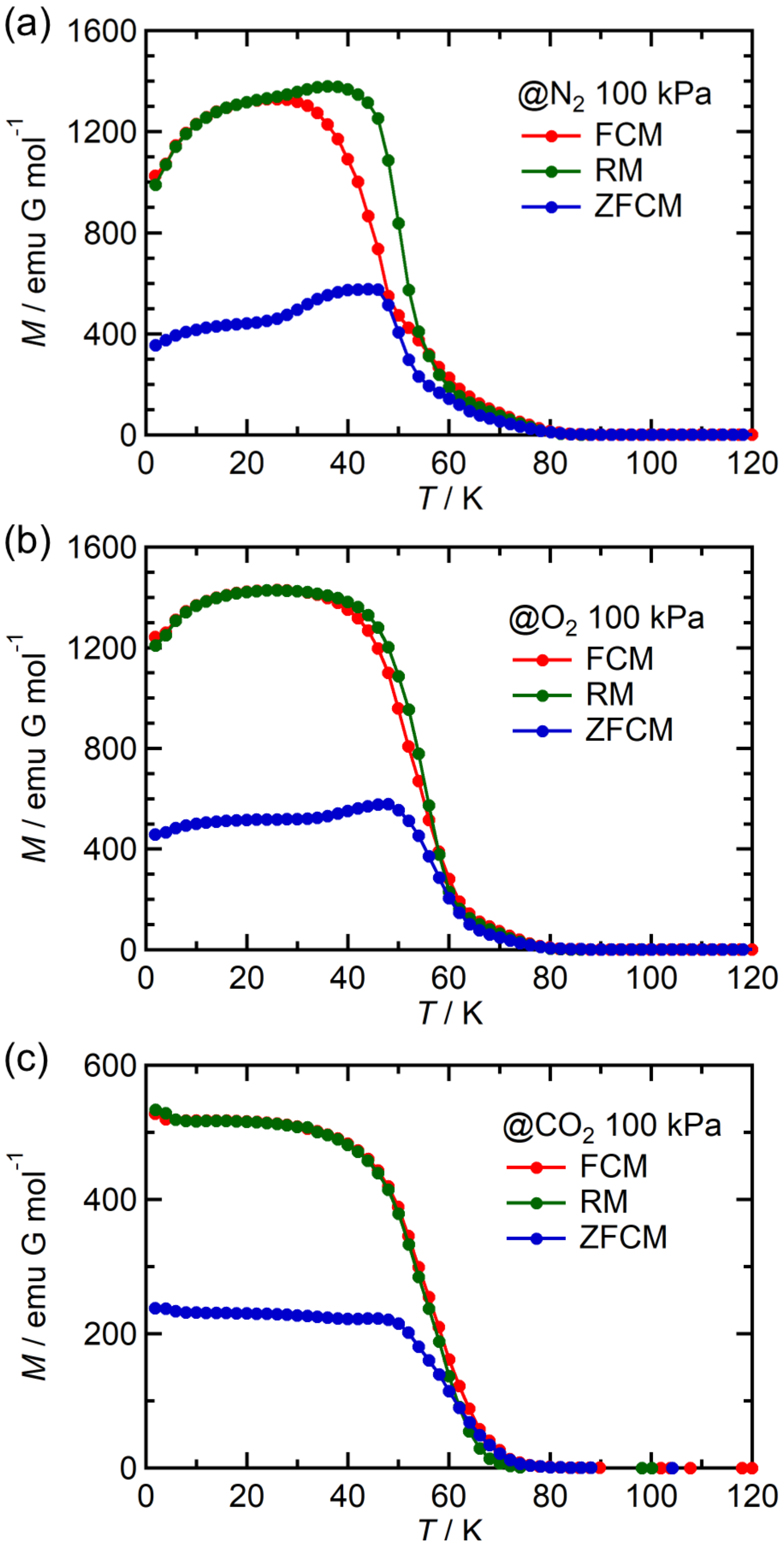

Fig. S14. FCM (red), RM (green), and zero-field-cooled magnetization (ZFCM, blue) curves at 5 Oe for **2⊃N₂** (a), **2⊃O₂** (b), and **2⊃CO₂** (c).

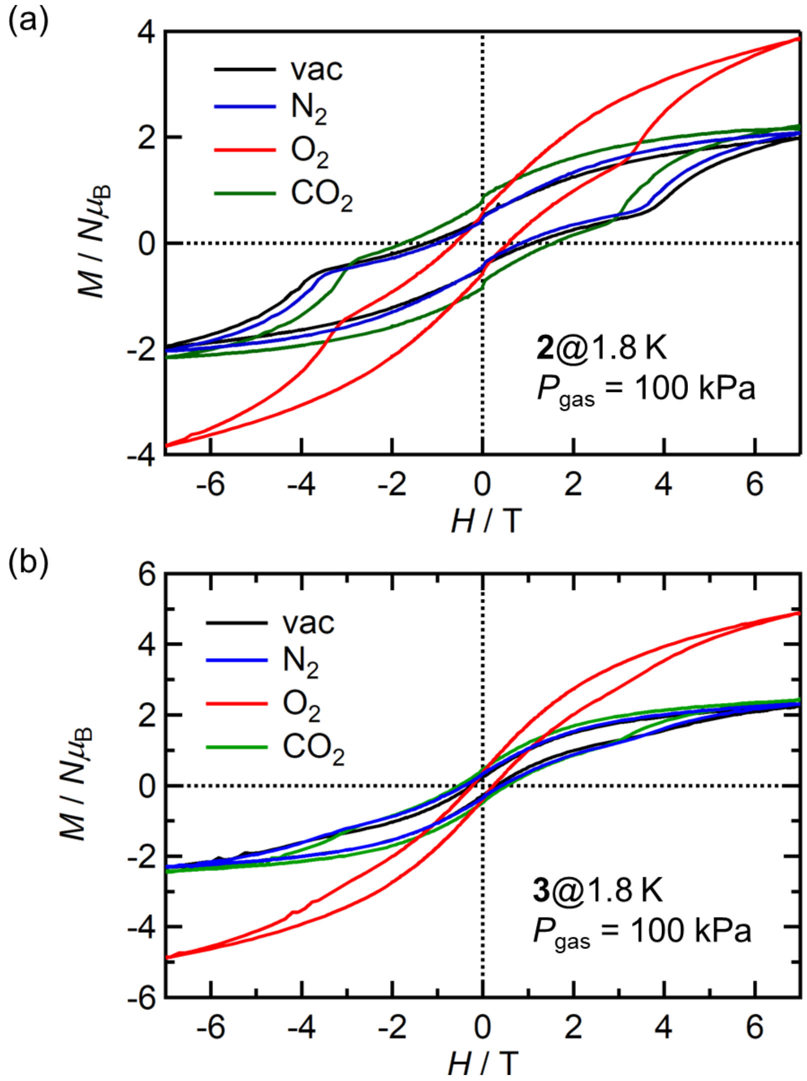

Fig. S15. (a) Magnetic hysteresis loops at 1.8 K for **2** measured under vacuum (black) and **2⊃N₂** (blue), **2⊃O₂** (red), and **2⊃CO₂** (green) under a 100 kPa gas atmosphere. (b) Magnetic hysteresis loops at 1.8 K for **3** measured under vacuum (black) and **3⊃N₂** (blue), **3⊃O₂** (red), and **3⊃CO₂** (green) under a 100 kPa gas atmosphere.

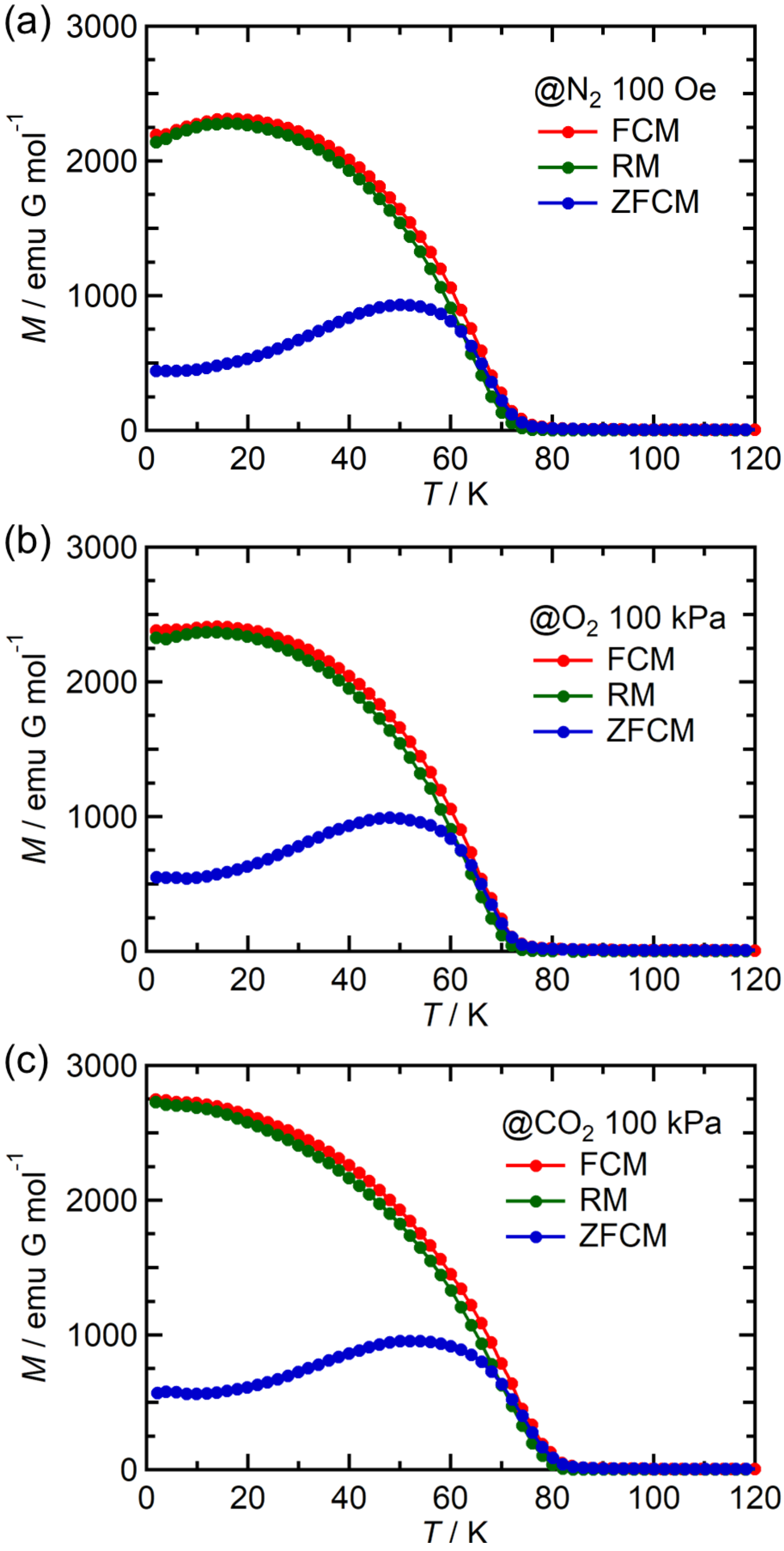

Fig. S16. FCM (red), RM (green), and zero-field-cooled magnetization (ZFCM, blue) curves at 100 Oe for **3⊃N₂** (a), **3⊃O₂** (b), and **3⊃CO₂** (c).

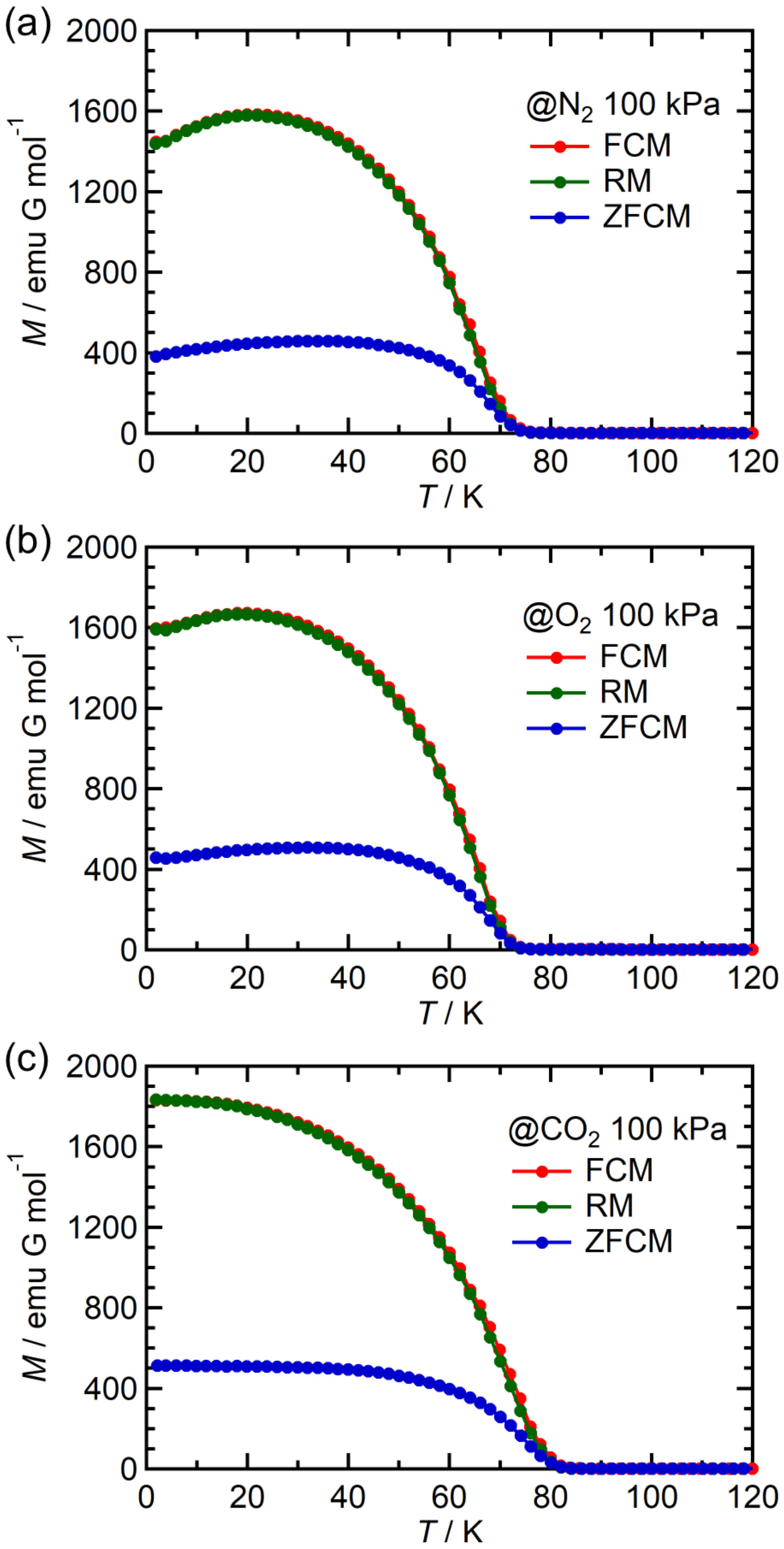

Fig. S17. FCM (red), RM (green), and zero-field-cooled magnetization (ZFCM, blue) curves at 5 Oe for **3⊃N₂** (a), **3⊃O₂** (b), and **3⊃CO₂** (c).

■ REFERENCES in SI

1 H. Miyasaka, N. Motokawa, R. Atsuumi, H. Kamo, Y. Asai and M. Yamashita, *Dalton Trans.*, 2011, **40**, 673−682.

2 H. Fukunaga, W. Kosaka, H. Nemoto, K. Taniguchi, S. Kawaguchi, K. Sugimoto and H. Miyasaka, *Chem. Eur. J.* 2020, **26**, 16755−16766.

3 G. A. Bain and J. F. Berry, *J. Chem. Educ.* 2008, **85**, 532−536.

4 S. Kawaguchi, M. Takemoto, K. Osaka, E. Nishibori, C. Moriyoshi, Y. Kubota, Y. Kuroiwa and K. Sugimoto, *Rev. Sci. Instrum.*, 2017, **88**, No. 085111.

5 F. Izumi and K. Mommma, *Solid State Phenom.*, 2007, **130**, 15−20.

6 V. Favre-Nicolin and R. Černý, *J. Appl. Cryst.*, 2002, **35**, 734−743.

7 A. L. Spek, *J. Appl. Cryst.*, 2003, **36**, 7−13.

8 K. Momma and F. Izumi, *J. Appl. Cryst.*, 2008, **41**, 653−658.

9 BIOVIA, Dassault Systems, Discovery Studio Visualizer v21.1.0, San Diego: Dassault Systems, 2020.

10 W. Kosaka, Z. Liu, J. Zhang, Y. Sato, A. Hori, R. Matsuda, S. Kitagawa and H. Miyasaka, *Nat. Commun.*, 2018, **9**, 5420.
